# Exploring the Chemical Space Accessed by Chiral Pool Terpenes. The (−)-Caryophyllene Oxide Paradigm

**DOI:** 10.1021/acs.orglett.4c00132

**Published:** 2024-03-29

**Authors:** Theodora Athanasiadou, Georgia G. Bagkavou, Polymnia Karagianni, Christos I. Stathakis

**Affiliations:** Department of Chemistry, Aristotle University of Thessaloniki, Thessaloniki 541 24, Greece

## Abstract

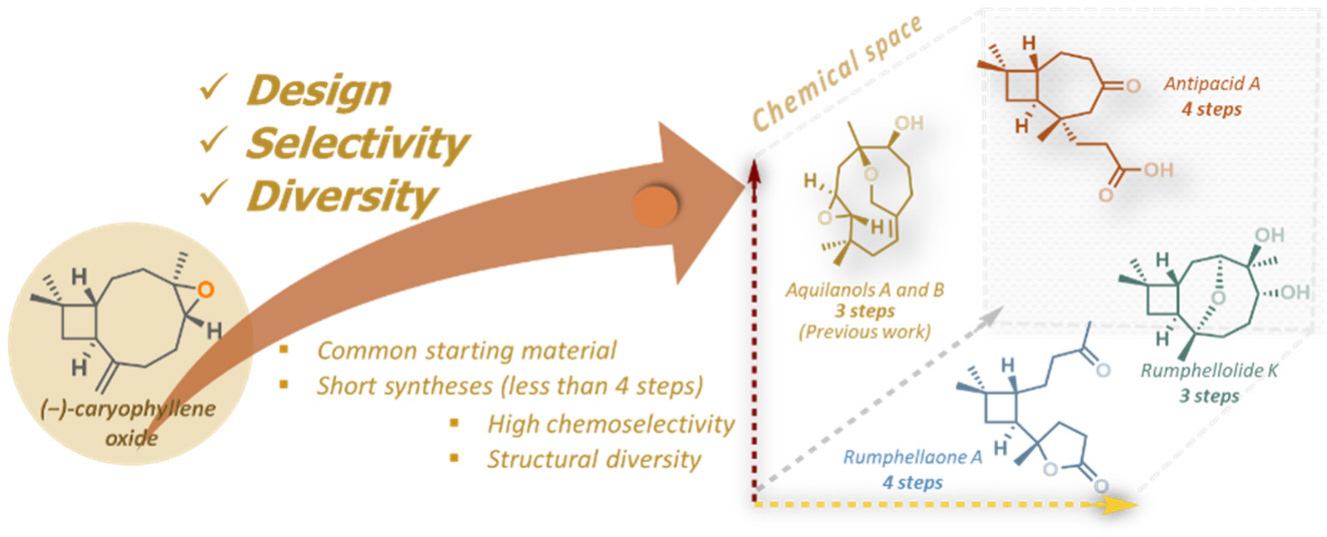

Terpenes represent a flourishing source of structural motifs that can be converted into several more complex architectures. Realization of such transformations in a concise and efficient manner adds great value to the starting material. Herein, we study the case of (−)-caryophyllene oxide and convert it into natural sesquiterpenoids (rumphellolide K, rumphellaone A, and antipacid A), thus expanding the chemical space accessed by its privilege structure. Our semisyntheses are short and rely on reagent-dictated stereo- and chemoselectivity.

Terpenes have a long history as starting materials in the construction of more complex natural products. Compounds such as (−)-citronellol, (+)-carvone, (−)-α-pinene, and (−)-isopulegol (Figure 1A) are easily accessible in large quantities from natural sources; therefore, they have been used as cheap feedstock material in elegant endeavors of the synthetic community.^1^ We believe that great potential to capitalize on privilege structures of chiral pool terpenes to gain rapid access to products of high added value still exists. Modern synthetic tools can enable short (fewer than five steps) and sophisticated paths to selected targets based on careful design and discovery of reagent-controlled selectivity.

**Figure 1:**
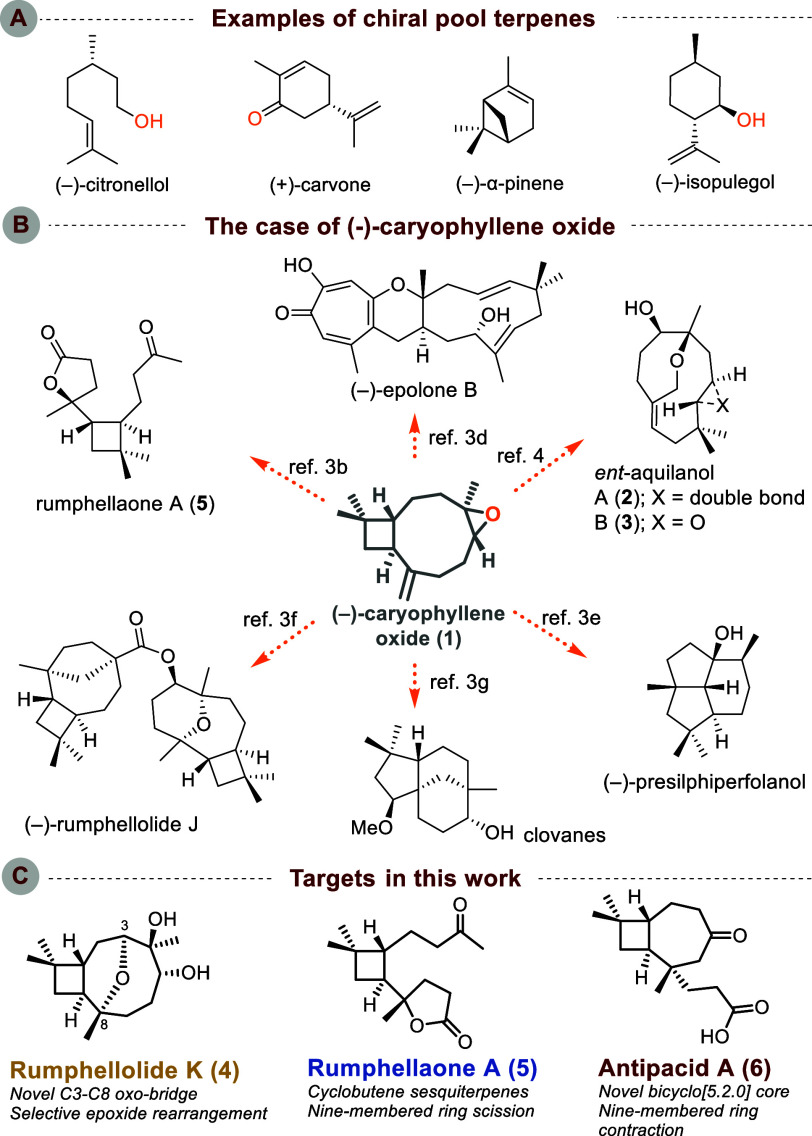
(A) Chiral pool terpenes as a starting point for advanced complexity. (B) Natural products synthesized from (−)-caryophyllene oxide (**1**). (C) Target molecules in this work.

(−)-Caryophyllene oxide (**1**) represents a special scaffold in terms of chemical reactivity mainly because of the restricted conformations of its medium-sized ring (Figure 1B).^2^ Several studies demonstrate the reagent-controlled transformation into unique rearranged products and/or natural products.^3^ We have recently presented its conversion to the enantiomers of two natural sesquiterpenoids, aquilanols A (**2**) and B (**3**), in three and four steps, respectively.^4^ In that work, (−)-caryophyllene oxide was converted into a monocyclic humulene-type derivative via a retro-cycloisomerization-induced ring expansion.

Exploring further the chemical space that can be accessed from (−)-caryophyllene oxide (**1**), we designed different strategies that capitalize on certain features of its structure, e.g., the epoxide moiety. In addition, selective manipulation of easily accessible derivatives of (**1**) allows for further transformations such as ring contractions that open the way to other families of natural products, such as antipacids [exemplified by antipacid A (**6**) (Figure 1C)].

First, we aimed to synthesize rumphellolide K (**4**), a recently isolated sesquiterpenoid from *Rumphella antipathies* that inhibits the generation of superoxide anions and the release of elastase by human neutrophils (Figure 1C).^5^ The proposed structure has the same bicyclic skeleton as that found in (−)-caryophyllene oxide, while an additional oxo bridge resides between C3 and C8 forming a tetrahydropyran. Although analogous ether bridges are known in caryophyllane-type natural products, the C3/C8 oxo bridge constitutes a novel type of connectivity. To access the target molecule, rumphellolide K (**4**), we envisioned an intramolecular opening of epoxide **7**, in a regioselective manner (Scheme 1). The latter could be derived from allylic alcohol **8** via stereoselective epoxidation. Finally, alcohol **8** can be considered as the rearranged epoxide found either in (−)-caryophyllene oxide itself or in its double-bond hydrated derivative.

**Scheme 1:**
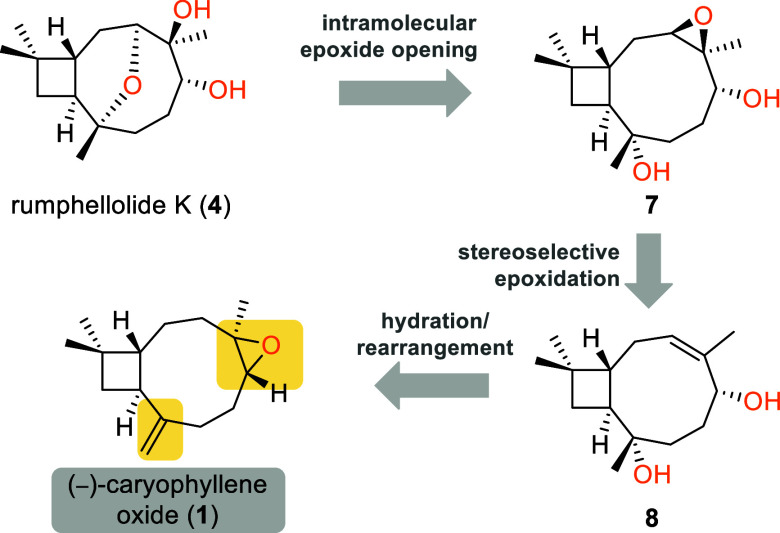
Retrosynthesis of Rumphellolide K (**4**) Employing (−)-Caryophyllene Oxide (**1**) as the Starting Material

Initial efforts to elaborate (−)-caryophyllene oxide to endocyclic allylic alcohol **9** were unproductive as inseparable mixtures of the desired alcohol with exocyclic one **9a** were afforded under any of the conditions tested (Scheme 2). To overcome this selectivity issue early in our synthetic plan, we proceed with the hydration of the exomethylenic double bond in caryophyllene oxide with the expectation that conformational changes would favor the desired isomer in the subsequent epoxide rearrangement. To perform the Markovnikov hydration of the double bond, we adopted a variant of Mukaiyama conditions reported by Studer and co-workers, using Fe(acac)_3_ as the catalyst and nitroarenes as the oxygen source.^6^ Significantly, under these modified conditions, the desired stereoisomer of tertiary alcohol **10** was produced almost exclusively, in accordance with the reported results. In contrast, under typical Mukaiyama hydration conditions, a 1.2:1 mixture of diastereomers was afforded.

**Scheme 2:**
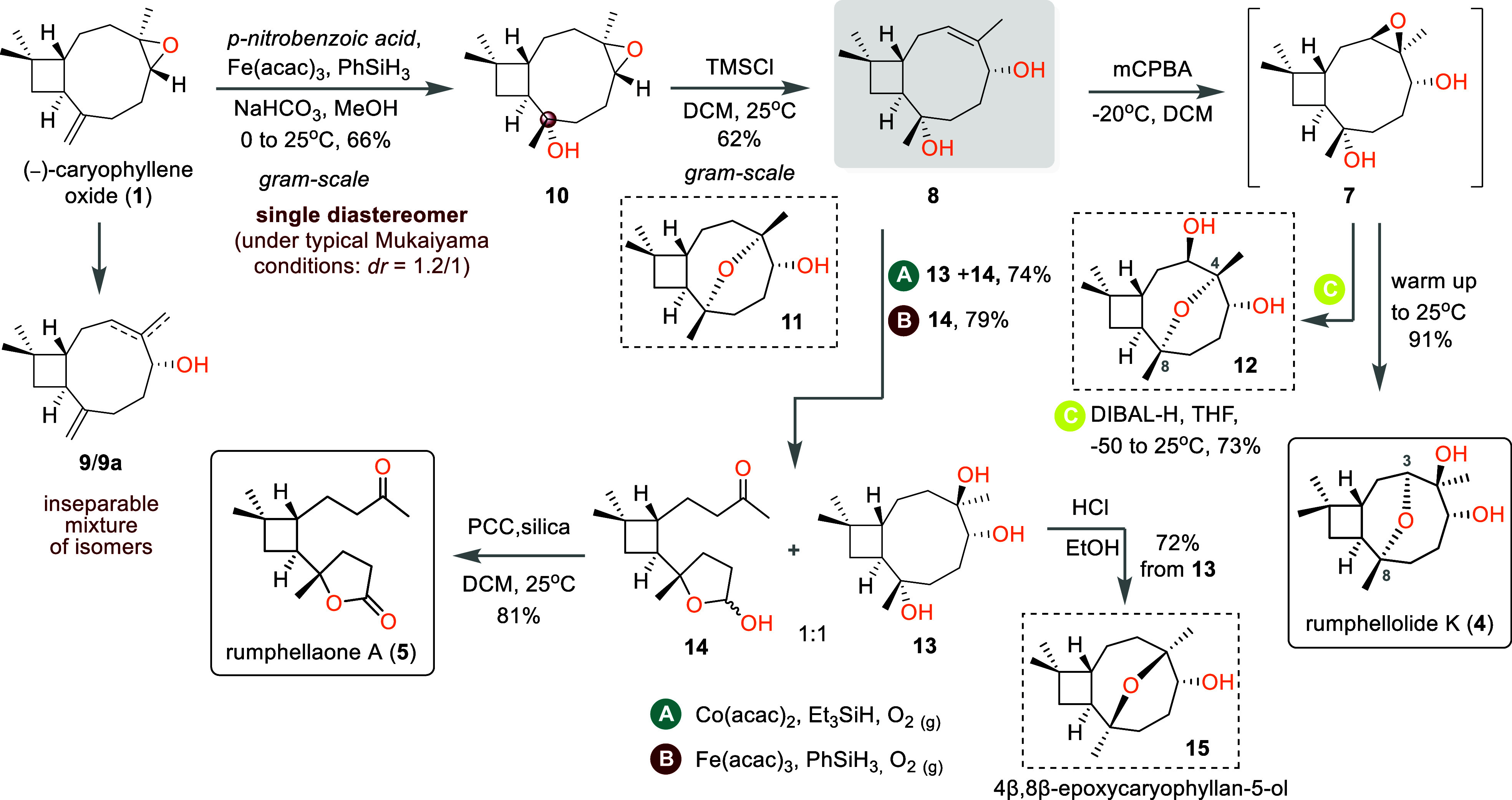
Synthesis of Rumphellolide K (**4**) and Rumphellaone A (**5**) from (−)-Caryophyllene Oxide (**1**)

Following highly diastereoselective hydration, we attempted the desired selective epoxide rearrangement to endocyclic allylic alcohol **8**. After much experimentation, we found that under strictly controlled acidic conditions, effected by a stoichiometric amount of TMSCl in commercial DCM, alcohol **10** was converted into allylic alcohol **8** in 62% optimized yield (Scheme 2), with oxo-bridged side product **11** accounting for the rest of the consumed starting material. Importantly, none of the isomeric exocyclic allylic alcohol (structure not shown) was observed, thus evading isolation problems similar to those we encountered with the **9**/**9a** mixture.

With access to critical advanced intermediate **8** secured, we proceeded with the epoxidation step to formulate cyclization precursor **7** (Scheme 2). Three-dimensional model studies indicated the desired stereoselectivity as the favorable one in a hydroxyl-directed epoxidation event owing to conformational restrictions. Indeed, using *m*-CPBA as the oxidizing agent at −20 °C afforded a single diastereomer with the correct configuration as evidenced by its conversion to the natural product. In fact, the formation of rumphellolide K was observed even during recording of NMR spectra of epoxide **7**, while it was dissolved in deuterated chloroform, or even by warming the epoxidation mixture to room temperature and stirring at the temperature for a further 30 min. This two-step cascade proved to be highly stereo- and regioselective, allowing the formation of only natural product rumphellolide K (**4**) in 91% combined yield.^7^

The three-step sequence described above not only provides rapid access to a more complex motif but also serves as a base for diversification, allowing the rational design of structurally distinct natural or unnatural architectures. Thus, the intramolecular epoxide opening in **7**, with the opposite regioselectivity compared to the one that leads to rumphellolide K, provides access to unknown *trans*-1,3-diol **12**, bearing a C4/C8 ether bridge (Scheme 2). This complementary regioselectivity was achieved by simply using DIBAL-H as the promoter.^8^

More indicative of the synthetic pluralism of this approach is the elaboration of key intermediate **8** to rumphellaone A [**5** (Figure 1)], a known cyclobutane derivative from the gorgonian coral *R. antipathies*.^9^ This 4,5-seco-caryophyllane sesquiterpenoid possesses a γ-lactone unprecedented in the caryophyllene-type natural products, while it is found to show cytotoxicity toward CCRF-CEM (human T-cell acute lymphoblastic leukemia) tumor cells (IC_50_ = 12.6 μg/mL). Thus, allylic alcohol **8** was subjected to standard Mukaiyama hydration conditions [Co(acac)_2_, Et_3_SiH, O_2_ (g)] to form triol **13** (Scheme 2).^10^ Surprisingly, along with the expected product, a less polar material was isolated from the reaction, as a mixture of diastereomers, the structure of which was attributed to lactols **14**, the C–C cleavage and spontaneous hemiacetal formation product (1:1 **13**:**14** ratio). To the best of our knowledge, this is the first example of a C–C cleavage of allylic alcohols triggered by homogeneous cobalt catalysis as under Mukaiyama hydration conditions. Via addition of NaIO_4_ to the reaction mixture upon consumption of the starting material, anomeric lactols **14** were isolated as the sole product in 78% yield via this two-step, one-pot process. Efforts to promote exclusively the C–C scission on allylic alcohol **8** led to the development of the optimized conditions involving Fe(acac)_3_, PhSiH_3_, and O_2_ in an ethanolic solution.^11^ Finally, oxidation of the lactol moiety in **14** proceeded promptly, using PCC as the oxidant, delivering rumphellaone A (**5**) in 81% yield. The physical and spectroscopic data of the isolated material are in good agreement with the reported data.^3b^ Furthermore, treatment of triol **13** with hydrochloric acid resulted in the complete conversion to the known natural product 4β,8β-epoxycaryophyllan-5-ol (**15**), a diastereomer of bridged ether **11**.

Having accomplished the synthesis of three intriguing natural sesquiterpenoids, one of them reported for the first time, we wanted to investigate other ways to selectively manipulate the bicyclic core of (−)-caryophyllene oxide (**1**). In particular, we wished to explore the possibility of achieving ring contraction of the nine-membered ring to a seven-membered one, to access another family of sesquiterpenes, antipacids A and B, recently isolated from *R. antipathies* (Scheme 3).^12^ More specifically, we targeted antipacid A [**6** (Figure 1)], bearing a novel bicyclo[5.2.0] core unprecedented in caryophyllane-type natural products.

**Scheme 3:**
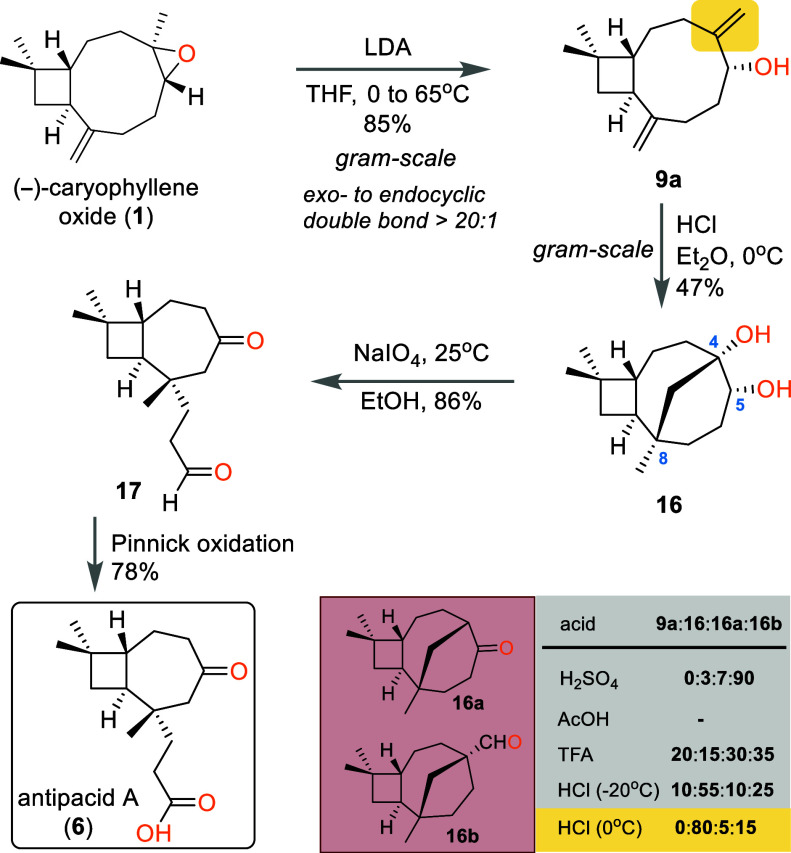
Synthesis of Antipacid A (**6**) from (−)-Caryophyllene Oxide (**1**)

For this purpose, the key starting material (**1**) was subjected to an epoxide rearrangement under basic conditions, to produce selectively exocyclic allylic alcohol **9a**. In sharp contrast to the attempted acidic conditions, in which only inseparable mixtures of **9** and **9a** were obtained, under the reported basic conditions (LDA, THF, reflux) the desired regioisomer was isolated almost exclusively (>20:1 **9a**:**9**) in high yield (Scheme 3).^13^ Next, acidic treatment of **9a** initiated a cationic reaction leading to the formation of tricyclic diol **16** accommodating a C4–C8 bridge. Among various acids tested to bring about this transformation, concentrated HCl in Et_2_O was the most favorable for the desired diol **16** distribution of products, the other two major side products being ketone **16a** and aldehyde **16b**.^14^ According to our design, a C4–C5 scission via a glycolic cleavage would reveal the seven-membered ring found in antipacid A. Indeed, treatment of diol **16** with NaIO_4_ delivered aldehyde **17** in 86% yield, which upon further oxidation using modified Pinnick conditions afforded antipacid A (**6**) in good yield.^15^

In summary, we have accomplished the stereoselective synthesis of three sesquiterpenoids, rumphellolide K (**4**, three steps), rumphellaone A (**5**, four steps), and antipacid A (**6**, four steps); the first and third compounds have been synthesized for the first time. In the course of our synthetic endeavors, selective access to similar, though structurally distinct, bridged ethers **11** and **12**, as well as natural 4β,8β-epoxycaryophyllan-5-ol (**15**), has been achieved. All of the synthesized compounds mentioned above, in addition to aquilanols A (**2**) and B (**3**) (Figure 1B), reported previously by our team,^4^ have originated from (−)-caryophyllene oxide (**1**), a privileged scaffold from terpenes’ chiral pool, and were all accessed in sequences shorter than four chemical steps. These results highlight the potential of such motifs to produce remarkable structural diversity in a concise and selective manner, thus affording significant added value to the feedstock material.

Finally, we have discovered conditions for performing direct Cα–Cβ cleavage of allylic alcohols, under mild conditions involving Fe(III) catalysis, a protocol that is currently under optimization, and the results will be reported in due course.

## Data Availability

The data underlying this study are available in the published article and its Supporting Information.

## Supporting Information Available

Full experimental details for the synthesis of all new compounds, their physical and spectroscopic data, and copies of ^1^H and ^13^C NMR spectra (PDF)
