# Supplementary material for: Exploring the Chemical Space Accessed by Chiral Pool Terpenes. The (−)-Caryophyllene Oxide Paradigm
Source: Org Lett. 2024 Mar 29;26(15):2897–901. doi: 10.1021/acs.orglett.4c00132 (PMC11187627; doi:10.1021/acs.orglett.4c00132)
Supplement: Supplementary file 1 — ol4c00132_si_001.pdf [file ol4c00132_si_001.pdf]

Supporting information

Exploring the Chemical Space Accessed by Chiral Pool Terpenes. The (−)-Caryophyllene Oxide Paradigm

Theodora Athanasiadou, Georgia G. Bagkavou, Polymnia Karagianni and Christos I. Stathakis*

Department of Chemistry, Aristotle University of Thessaloniki, Thessaloniki, 541 24, Greece

*Email: cstathakis@chem.auth.gr

Table of contents

General information ………………………………………………………………………………………………… S1

Detailed experimental procedures……………………………………………………………………………. S2

Synthesis of rumphellolide K (**4**)……………………………………………………………………………… S2

Synthesis of rumphellaone A (**5**)…………………………………………………………………………….. S6

Synthesis of antipacid A (**6**)………………………………………………………………………….……. S10

References…………………………………………………………………………..…………………………….. S13

Copies of NMR spectra……………………………………………………………………………………………. S14

General information

All reagents are commercially available and were used without further purification unless otherwise stated. Solvents were dried by standard methods.[1] Reactions were monitored by thin-layer chromatography (TLC) carried out on Merck silica gel 60 F254 glass plates (0.25 mm) using UV light as visualizing agent and Seebach solution as developing agent.[2] Column chromatography was performed silica gel (0.040 – 0.063 mm, 230 – 400 mesh ASTM) from Merck. Silica gel was neutralized with 1% v/v Et_3N in cyclohexane and used in all indicative cases where compounds were sensitive to acidic conditions. Melting points were determined with a Koffler hot-stage microscope.

NMR spectra were recorded on an Agilent 500 MHz spectrometer or a Bruker Avance III 300 MHz spectrometer and were calibrated using solvent residual peaks as an internal reference. The following abbreviations are used to designate multiplicities: *s* = singlet, *d* = doublet, *t* = triplet, *q* = quartet, *m* = multiplet, *br* = broad, *dd* = doublet of doublets, *dd* = doublet of triplets, *AB* = AB type of spin system. Optical rotations were recorded on a Perkin-Elmer Model 343 polarimeter at 589 nm. HRMS spectra were recorded on a Brucker® Maxis Impact QTOF spectrometer.

Detailed experimental procedures.

Synthesis of rumphellolide K

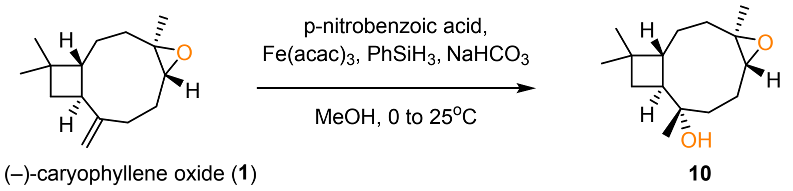

(1*R*,4*R*,6*R*,9*S*,10*S*)-4,9,12,12-Tetramethyl-5-oxatricyclo[8.2.0.0^{4,6}]dodecan-9-ol (10): A 5ml flame dried round bottom flask flashed with argon, was charged sequentially with (–)-caryophyllene oxide (**1**) (220 mg, 1.0 mmol, 1.0 equiv), nitrobenzoic acid (217 mg, 1.3 mmol, 1.3 equiv), $NaHCO_3$ (168 mg, 2.0 mmol, 2.0 equiv), $Fe(acac)_3$ (9 mg, 0.025 mmol, 2,5 mol%), and MeOH (8.0 ml). The mixture was cooled down to 0°C and $PhSiH_3$ (0.37 ml, 3.0 mmol, 3.0 equiv) was added dropwise and a red color was developed. The reaction mixture was slowly warmed to room temperature and stirred there for 3 h, during which time the color of the reaction turned from dark red to brown. Then, solvent was removed, and the residue was purified by flash column chromatography (cyclohexane:EtOAc = 8:1) using neutralized silica gel (1% v/v Et_3N in cyclohexane) to provide 156 mg of product (66% yield) as a white solid. The spectroscopic data of **10** are consisted with the reported in the literature.[3]

mp 60 – 63°C, $[a]^{25}_D$= -106 (*c* 0.19, $CHCl_3$); [lit$[a]^{30}_D$= -105.2 (*c* 4.63, $CHCl_3$)].[4] **^{1}H NMR** (500 MHz, C_6D_6) δ 2.52 (dd, *J* = 9.6, 5.7 Hz, 1H), 1.96 – 1.88 (m, 1H), 1.86 (dt, *J* = 12.9, 3.7 Hz, 1H) 1.78 (q, *J* = 9.1 Hz, 1H), 1.67 (ddd, *J* = 13.9, 5.7, 2.9 Hz, 1H), 1.55 – 1.41 (m, 3H), 1.34 – 1.23 (m, 5H), 1.08 (s, 3H), 0.99 (s, 3H), 0.80 (s, 3H), 0.79 (s, 3H); **^{13}C NMR** (126 MHz, C_6D_6) δ 74.2, 60.2, 58.5, 52.5, 47.2, 40.7, 40.5, 35.5, 31.8, 30.1, 29.0, 25.0, 23.3, 20.7, 16.5.

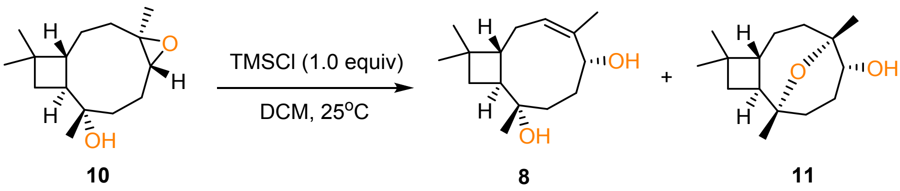

(1*S*,2*S*,5*R*,9*R*,*Z*)-2,6,10,10-tetramethylbicyclo[7.2.0]undec-6-ene-2,5-diol (8) and (1*S*,2*S*,5*R*,8*S*,9*R*)-1,4,4,8-tetramethyl-12-oxatricyclo[6.3.1.0^{2,5}]dodecan-9-ol (11): Into a round bottom flask filled with argon, TMSCl (79 µL, 0.629 mmol, 1.0 equiv) was added

dropwise at 0°C to a solution of epoxide **10** (150 mg, 0.629 mmol, 1.0 equiv) in DCM (15 ml). The cooling bath was removed and stirring maintained for 10 min further at room temperature. At this point, the reaction mixture was carefully quenched with H_2O (10 ml). Organic phase was separated and the aqueous one was extracted with EtOAc (2 x 5 ml). The organic layers were combined, dried over anhydrous Na_2SO_4 and evaporated under reduced pressure. Purification by flash column chromatography (cyclohexane:EtOAc = 8:1 to 2:1) led to the isolation of diol **8** (white solid, 93 mg, 62%) and the ether **11** (white solid, 51 mg, 35%).

Diol 8: mp 102 – 105°C, $[a]^{25}_D$ = -81.8 (*c* 0.083 M, $CHCl_3$). **^{1}H NMR** (500 MHz, $CDCl_3$) δ 5.44 (t, *J* = 8.1 Hz, 1H), 4.39 (dd, *J* = 9.4, 3.1 Hz, 1H), 2.25 – 2.15 (m, 2H), 1.99 (ddd, *J* = 14.9, 8.0, 4.4 Hz, 1H), 1.92 – 1.80 (m, 2H), 1.78 – 1.68 (m, 2H), 1.76 (s, 3H), 1.65 – 1.46 (m, 3H), 1.18 (s, 3H), 1.01 (s, 3H), 0.99 (s, 3H); **^{13}C NMR** (126 MHz, $CDCl_3$) δ 138.9, 124.8, 73.7, 72.2, 46.6, 42.4, 39.5, 33.7, 33.3, 30.4, 30.2, 25.6, 23.8, 23.6, 18.1. **HRMS** (ESI) m/z $[M+Na]^+$ calculated for $C_{15}H_{26}NaO_2^+$: 261.1825, found: 261.1825.

Ether 11: mp 61 – 64°C, $[a]^{25}_D$ = -65.4 (*c* 0.043 M, $CHCl_3$). **^{1}H NMR** (500 MHz, $CDCl_3$) δ 3.52 – 3.48 (m, 1H), 2.13 – 2.05 (m, 1H), 1.99 (td, *J* = 11.6, 7.6 Hz, 1H), 1.91 – 1.83 (m, 2H), 1.79 – 1.64 (m, 3H), 1.61 – 1.40 (m, 4H), 1.21 (s, 3H), 1.17 (t, *J* = 10.6 Hz, 1H) 1.05 (s, 3H), 1.01 (s, 3H), 1.00 (s, 3H); **^{13}C NMR** (126 MHz, $CDCl_3$) δ 77.9, 73.7, 70.4, 48.3, 47.5, 39.1, 36.0, 35.8, 30.5, 29.7, 28.7, 25.9, 24.9, 23.3, 20.5.

NMR spectra are in good agreement with the reported ones.[5]

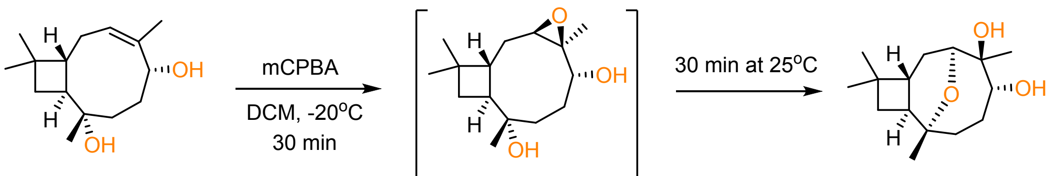

8 **7** rumphellolide K (**4**)

(1*R*,3*R*,5*S*,6*R*,9*S*,10*S*)-5,9,12,12-tetramethyl-4-oxatricyclo[8.2.0.0^{3,5}]dodecane-6,9-diol (7) and rumphellolide K (4): A round bottom flask, filled with argon, was charged with a solution of compound **8** (190 mg, 0.80 mmol, 1.0 equiv) in 10 ml DCM. After cooling at -20°C, mCPBA (70% purity; 256 mg, 1.04 mmol, 1.3 equiv) was added and the mixture was stirred for 30 min, at which point full conversion of starting material to a slightly more polar spot, epoxide **7**, was observed as evidenced by TLC. Compound **7** could be isolated (see work-up below) and fully characterized. Alternatively, the reaction mixture could be warmed up to ambient temperature and stir there for 30 – 60 min further, period during which epoxide **7** was

converted to rumphellolide K (**4**). The reaction was then quenched with 5% w/v *aq*. $Na_2S_2O_5$ (5 ml) and organic phase was separated. The aqueous one was extracted twice with EtOAc (2 x 5 ml), the organic layers were combined, dried over anhydrous Na_2SO_4 and evaporated under reduced pressure. The residue was purified by column chromatography (cyclohexane:EtOAc = 2:1) and natural product rumphellolide K (**4**) was obtained in 91% combined yield, as a colorless oil.
As mentioned above, epoxide **7** could be isolated by quenching the reaction while still at -20°C following the same work-up protocol. After careful purification on neutralized silica (1% Et_3N in cyclohexane), epoxide **7** was obtained in 95% yield as colorless oil.

Epoxide 7: $[a]^{25}_{D}$= -4.9 (*c* 0.014, $CHCl_3$). **^{1}H NMR** (500 MHz, $CDCl_3$) δ 3.38 (d, *J* = 9.2 Hz, 1H), 3.17 (dd, *J* = 11.6, 4.4 Hz, 1H), 2.31 (q, *J* = 9.7 Hz, 1H), 2.09 – 1.91 (m, 3H), 1.83 – 1.77 (m, 1H), 1.67 (d, *J* = 9.7 Hz, 2H), 1.55 (dd, *J* = 15.4, 12.3 Hz, 1H), 1.41 (s, 3H), 1.40 – 1.25 (m, 2H), 1.17 (s, 3H), 1.11 (s, 3H), 1.00 (s, 3H); **^{13}C NMR** (126 MHz, $CDCl_3$) δ 77.3, 73.2, 63.9, 63.7, 43.1, 39.6, 39.2, 34.1, 33.6, 30.7, 27.0, 26.3, 25.3, 25.0, 16.0. **HRMS** (ESI) m/z $[M+Na]^+$ calculated for $C_{15}H_{26}NaO_3^+$: 277.1774, found: 277.1774.

Rumphellolide K (4): **^{1}H NMR** (500 MHz, $CDCl_3$) δ 4.11 (dd, *J* = 10.6, 4.5 Hz, 1H), 3.88 – 3.83 (m, 1H), 2.14 (br, OH, 1H), 2.11 – 2.00 (m, 2H), 1.92 – 1.85 (m, 1H), 1.85 – 1.77 (m, 1H), 1.74 – 1.67 (m, 1H), 1.68 – 1.60 (m, 1H), 1.57 – 1.50 (m, 2H), 1.47 (dd, *J* = 10.1, 7.7 Hz, 1H), 1.27 (s, 3H), 1.15 (t, *J* = 10.5 Hz, 1H), 1.05 (s, 3H), 1.02 (s, 3H), 1.01 (s, 3H); **^{13}C NMR** (126 MHz, $CDCl_3$) δ 81.4, 78.6, 74.0, 69.4, 48.1, 40.7, 35.7, 35.3, 34.5, 30.3, 29.7, 28.4, 25.0, 23.3, 21.2. **HRMS** (ESI) m/z $[M+Na]^+$ calculated for $C_{15}H_{26}NaO_3^+$: 277.1774, found: 277.1774.

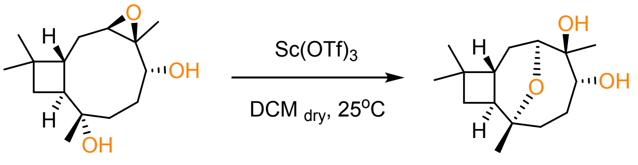

7 rumphellolide K (**4**)

Epoxide **7** could itself deliver rumphellolide K (**4**) under acidic conditions. Various acids were tested (see Table S1), that all afforded exclusively the same product in high yields. Experimental details for the most effective one, scandium (III) triflate, are described below:

Epoxide **7** (20 mg, 0.08 mmol, 1.0 equiv) was charged in a round bottom flask (filled with argon) and dissolved in 1.6 ml dry DCM. Then, $Sc(OTf)_3$ (4 mg, 0.008 mmol, 10 mol%) was

added and the reaction mixture was stirred for 30 min. TLC monitoring showed completion of the reaction and the formation of a new spot. The mixture was quenched with H_2O (2 ml) and extracted twice with DCM (2 x 3 ml). The organic layers were combined, dried over anhydrous Na_2SO_4 and evaporated under reduced pressure. After purification by column chromatography (cyclohexane:EtOAc = 2:1), 19 mg of rumphellolide K (96% yield) were obtained as colorless oil.

Table S1: Conditions used for the last step synthesis of rumphellolide K

Entry	Reagent	Solvent	T [°C]	Yield [%][a]
**1**	BF_3•OEt_2	DCM	25	90
**2**	**$Sc(OTf)_3$**	**DCM**	**25**	**96**
**3**	TFA	DCM	0	92
**4**	K_2CO_3	$CHCl_3$	25-50	No Reaction

[a] Yields refer to isolated products; OTf = trifluoromethanosulfonate, TFA = trifluoroacetic acid.

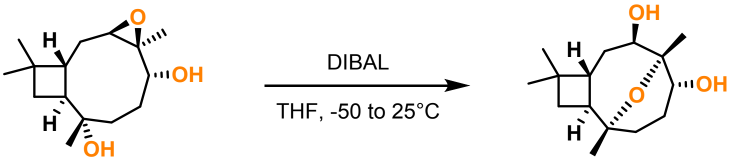

7 12

(1*S*,2*S*,5*R*,7*R*,8*R*,9*R*)-1,4,4,8-tetramethyl-12-oxatricyclo[6.3.1.0^{2,5}]dodecane-7,9-diol (12): In a vial filled with nitrogen, epoxide **7** (7 mg, 0.028 mmol) was dissolved in dry THF (0.4ml). DIBAL (56 µl, 0.056 mmol, 2.0 equiv) was added at -50°C. Then, the temperature was raised gradually to 0°C and mixture was stirred there for 4 hours before it was further warmed to 25°C and stirred for another 3 hours. After reaction completion, H_2O was added carefully dropwise, and the reaction mixture was extracted with EtOAc (2 x 3 ml). The combined organic phases were dried over anhydrous Na_2SO_4, filtered, and concentrated in vacuo to remove solvents. Crude ether was purified by column chromatography using cyclohexane/EtOAc (1:1 v/v) as the eluent to obtain 5.2 mg of a white amorphous solid (yield 73%).

$[a]^{25}_{D}$= +69 (c 0.09, $CHCl_3$). **^{1}H NMR** (500 MHz, $CDCl_3$) δ 4.14 (dd, *J* = 9.5, 4.9 Hz, 1H), 3.88 (m, 1H), 2.13 – 1.96 (m, 2H), 1.88 (q, *J* = 10.7 Hz, 1H), 1.71 (td, *J* = 12.1, 5.4 Hz, 1H), 1.70 – 1.65 (m, 2H), 1.62 – 1.51 (m, 2H), 1.48 (t, *J* = 8.9 Hz, 1H), 1.28 (s, 3H), 1.16 (t, *J* = 10.5 Hz, 1H), 1.06 (s, 3H), 1.03 (s, 3H), 1.01 (s, 3H); **^{13}C NMR** (500 MHz, $CDCl_3$) (126 MHz, $CDCl_3$) δ 81.4, 78.6, 74.0, 69.4, 48.2, 40.7, 35.7, 35.3, 34.6, 30.3, 29.7, 28.4, 25.0, 23.3, 21.2 **HRMS** (ESI) m/z $[M+Na]^+$ calculated for $C_{15}H_{26}NaO_3^+$: 277.1774, found: 277.1781.

Synthesis of rumphellaone A

The unexpected glycolic cleavage on allylic alcohol **8** towards lactole **14** under Mukaiyama conditions was further studied to probe the role of catalyst, as well as other parameters, and to improve selectivity in favor of the unexpected (albeit desired) product **14**, versus the expected hydration product, triol **13**. The results are summarized on the table below:

Table S2: Trials on the attempted sequential Mukaiyama hydration – glycolic cleavage[a]

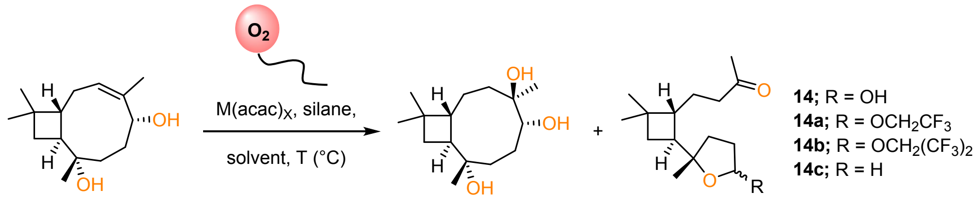

8 **13**

Entry	**Catalyst**	**Silane**	**Solvent**	**Temp. (°C)**	**Product**
**1**	Co(acac)$_2$	Et$_3$SiH	EtOH	25	**13**:**14** = 42:58
**2**	Co(acac)$_2$	Et$_3$SiH	MeOH	25	**13**:**14** = 47:53
**3**	Co(acac)$_2$	Et$_3$SiH	TFE	25	**13**:**14a** = 38:62
**4**	Co(acac)$_2$	Et$_3$SiH	HFIP	25	**13**:**14b**:**14c** = 35:42:27
**5**	Co(acac)$_2$	Et$_3$SiH	EtOH[b]	25	**13**:**14** = 44:56
**6**	Co(acac)$_2$[c]	Et$_3$SiH	EtOH	25	**13**:**14** = 51:49
**7**	Co(acac)$_2$	Et$_3$SiH	HFIP	0	**13**:**14b** = 38:62
**8**	Fe(acac)$_3$	PhSiH$_3$	EtOH	25	**14** (75%)
**9**	Fe(acac)$_3$	PhSiH$_3$[d]	EtOH	25	**14** (78%)
**10**	Fe(acac)$_3$	Et$_3$SiH	EtOH	25	No reaction
**11**[e]	Fe(acac)$_3$	PhSiH$_3$	EtOH	25	**13**:**14** = 58:42
**12**[f]	Fe(acac)$_3$	PhSiH$_3$	EtOH	25	**14** (79%)

[a] Standard conditions involved 20 mol% of catalyst and 3.0 equiv of silane and the reaction run in EtOH at 0.2 M concentration; The ratio of products is estimated by NMR [b] the concentration of the reaction was adjusted at 0.04 M; [c] 50 mol% Co(acac)$_2$ was used; [d] 2.0 equiv of PhSiH$_3$ was used; [e] para-nitrobenzoic acid was used as the oxygen atom source; [f] 10 mol% Fe(acac)$_3$ and 2.0 equiv of PhSiH$_3$ were used.

Triol **13** was isolated as the minor product under typical Mukaiyama conditions using Co(acac)$_2$ and Et$_3$SiH as the catalytic system:

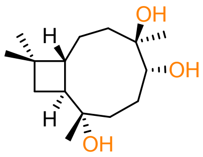

13

(1*S*,2*S*,5*R*,6*R*,9*R*)-2,6,10,10-tetramethylbicyclo[7.2.0]undecane-2,5,6-triol (13): white solid, mp=60-63°C, $[a]^{25}_{D}$= -22 (*c* 0.012, $CHCl_3$). **^{1}H NMR** (500 MHz, $CDCl_3$) δ δ 3.68 (dt, *J* = 10.4, 2.6 Hz, 1H), 2.23 (d, *J* = 2.6 Hz, 1H, OH), 2.06 (q, *J* = 9.4 Hz, 1H), 2.05 (s, 1H, OH), 1.88 – 1.64 (m, 7H), 1.63 – 1.53 (m, 3H), 1.43 – 1.33 (m, 1H), 1.26 (s, 3H), 1.20 (bs, 1H, OH), 1.13 (s, 3H), 1.01 (s, 3H), 0.96 (s, 3H); **^{13}C NMR** (126 MHz, $CDCl_3$) δ 75.4, 74.3, 73.5, 46.6, 45.0, 40.0, 37.0, 33.5, 32.8, 30.6, 29.5, 23.6, 22.1 , 22.0, 21.6. **HRMS** (ESI) m/z $[M+Na]^+$ calculated for $C_{15}H_{28}NaO_3^+$: 279.1931, found: 279.1934.

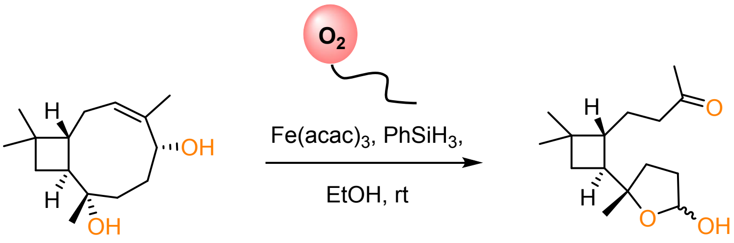

8 **14**

4-((1*R*,4*S*)-4-((2*S*)-5-hydroxy-2-methyltetrahydrofuran-2-yl)-2,2-dimethylcyclobutyl)butan-2-one (14): Into a microwave vial $Fe(acac)_3$ (13.4 mg, 0.038 mmol, 10 mol %) was added followed by a solution of diol **8** (90 mg, 0.38 mmol, 1.0 equiv) in 1.5 mL EtOH. The vial was sealed with a crimp cap with septum, and the mixture was bubbled with O_2(g) for 10 minutes and then a solution of $PhSiH_3$ (94 µL, 0.76 mmol, 2.0 equiv) in 0.5 mL EtOH was added over 30 min. The reaction mixture was stirred at room temperature for 4 h before the solvent was evaporated in vacuo. Then, H_2O was added, and the reaction mixture was extracted twice with EtOAc (2 x 5 ml). The combined organic phases were washed with brine, dried over anhydrous Na_2SO_4, filtered, and concentrated under vacuo. The residue was purified by column chromatography using cyclohexane:EtOAc = 2:1 as the eluent to obtain 74 mg of lactols **14** (inseparable 1:1 mixture of diastereomers) as a colorless oil (79% yield).

^{1}H NMR (500 MHz, $CDCl_3$, as a mixture) δ 5.51 (t, *J* = 2.6 Hz, 1H), 5.45 (d, J = 4.1 Hz, 1H), 3.54 (bs, 1H, OH), 3.38 (bs, 1H, OH), 2.43 – 2.30 (m, 4H), 2.10 (s, 3H), 2.09 (s, 3H), 2.07 – 2.01 (m, 2H), 1.97 – 1.83 (m, 4H), 1.83 – 1.73 (m, 4H), 1.71 – 1.42 (m, 9H), 1.32 (t, *J* = 10.2 Hz, 1H), 1.27 (s, 3H), 1.04 (s, 3H), 1.02 (s, 3H), 1.01 (s, 6H), 0.98 (s, 3H); **^{13}C NMR** (126 MHz, $CDCl_3$, as a

mixture) δ 209.6, 209.0, 99.0, 98.0, 86.0, 85.6, 45.1, 44.7, 44.6, 44.1, 42.3, 42.1, 34.8, 34.6, 34.1, 33.1, 33.0, 32.9, 32.7, 31.2, 31.1, 30.9, 29.9, 29.8, 27.7, 25.4, 25.4, 25.3, 22.7, 22.5. **HRMS** (ESI) m/z $[M+Na]^+$ calculated for $C_{15}H_{26}NaO_3^+$: 277.1774, found: 277.1777.

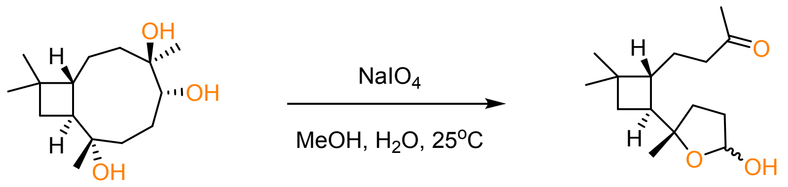

13 **14**

4-((1*R*,4*S*)-4-((2*S*)-5-hydroxy-2-methyltetrahydrofuran-2-yl)-2,2-dimethylcyclobutyl)butan-2-one (14): In a round bottom flask triol **7** (32 mg, 0.13 mmol) was dissolved in MeOH/H_2O (1.5 mL, 9:1 v/v), $NaIO_4$ (33 mg, 0.156 mmol, 1.2 equiv) was added and the reaction mixture was stirred for 1 h at 25°C. After completion, as evidenced by TLC analysis, MeOH was evaporated under vacuo, H_2O was added (5 ml), and the reaction mixture was extracted twice with EtOAc (2 x 5 ml). The combined organic phases were washed with brine, dried with anhydrous Na_2SO_4, filtered, and concentrated under vacuo. The residue was purified by column chromatography using cyclohexane:EtOAc = 2:1 as the eluent to obtain 27 mg of pure lactols **14** (as an inseparable mixture of diastereomers in 1:1 ratio) as colorless oil (84% yield).

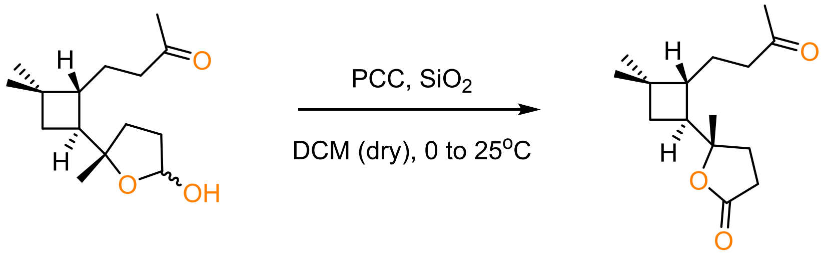

14 rumphellaone A (**5**)

Rumphellaone A (9): In a vial filled with argon, the mixture of lactols **14** (16.5 mg, 0.065 mmol) was dissolved in 1.5 ml of dry DCM. PCC (20 mg, 0.097 mmol, 1.5 equiv) and silica (25 mg) were added at 0°C. Then, the mixture was warmed up to 25°C and stirred there for 40 min. After full consumption of starting material, evidenced by TLC, the reaction was filtered through silica gel and rinsed with Et_2O. The filtrate was concentrated under vacuo to remove volatiles. Crude reaction mixture was purified by column chromatography using

cyclohexane:EtOAc = 3:1 as the eluent to obtain rumphellaone A (**5**) as a white amorphous solid (13.3 mg, 81%).

$[a]^{25}_{D}$= +66.7 (*c* 0.1, $CHCl_3$); [lit$[a]^{25}_{D}$= +65.6 (*c* 1.1, $CHCl_3$)],[6] **^{1}H NMR** (500 MHz, $CDCl_3$) δ 2.65 – 2.55 (m, 1H), 2.52 (ddd, *J* = 18.1, 10.0, 4.9 Hz, 1H), 2.35 (t, *J* = 7.7 Hz, 2H), 2.11 (s, 3H), 2.07 – 1.98 (m, 2H), 1.90 – 1.81 (m, 2H), 1.67 – 1.61 (m, 2H), 1.55 (t, *J* = 9.9 Hz, 1H), 1.41 (t, *J* = 10.4 Hz, 1H), 1.30 (s, 3H), 1.04 (s, 3H), 1.01 (s, 3H); **^{13}C NMR** (126 MHz, $CDCl_3$) δ 208.6, 176.9, 87.2, 44.5, 44.2, 42.0, 33.5, 33.0, 30.9, 30.6, 29.9, 29.1, 25.1, 24.8, 22.5. **HRMS** (ESI) m/z $[M+Na]^+$ calculated for $C_{15}H_{24}NaO_3^+$: 275.1618, found: 275.1620.

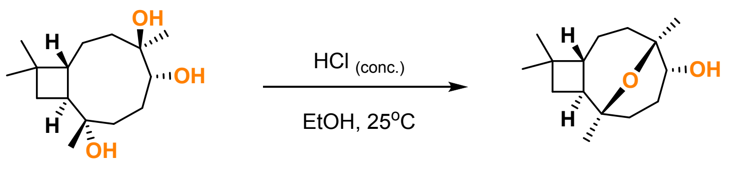

13 4*β*,8*β*-epoxycaryophyllan-5-ol (**15**)

***4β,8β*-Epoxycaryophyllan-5-ol (15)**: In a vial triol **13** (8 mg, 0.031 mmol) was dissolved in EtOH (0.5ml) and then was added concentrated HCl (2-3 drops) and the reaction mixture was stirred for 10 min at 25°C. After reaction completion, EtOH was evaporated in vacuo, H_2O was added, and the reaction mixture was extracted with EtOAc (2 ml). The organic phase was dried over anhydrous Na_2SO_4, filtered, and concentrated in vacuo. The crude mixture was purified by column chromatography using cyclohexane/EtOAc (4:1 v/v) as the eluent to obtain pure **15** as colorless oil (5.3 mg, 72% yield).

$[a]^{25}_{D}$= -83 (c 0.17, $CHCl_3$); [lit$[a]^{26}_{D}$= -70 (*c* 0.03, $CHCl_3$)].[7] **^{1}H NMR** (300 MHz, $CDCl_3$) δ 3.47 (dd, *J* = 11.3, 4.8 Hz, 1H), 2.54 (dt, *J* = 11.6, 9.8 Hz, 1H), 2.15 – 2.04 (m, 1H), 1.97 (dd, *J* = 13.0, 6.6 Hz, 1H), 1.88 (dd, *J* = 13.9, 4.2 Hz, 1H), 1.81 – 1.69 (m, 1H), 1.67 – 1.63 (m, 1H), 1.62 – 1.59 (m, 2H), 1.45 (t, *J* = 8.9 Hz, 2H), 1.35 (t, *J* = 10.1 Hz, 1H), 1.32 – 1.27 (m, 1H), 1.21 (s, 3H), 1.05 (s, 3H), 1.02 (s, 3H), 1.01 (s, 3H). **^{13}C NMR** (300 MHz, $CDCl_3$) (75 MHz, $CDCl_3$) δ 76.7, 75.5, 72.7, 42.4, 36.2, 36.2, 35.2, 34.4, 30.5, 29.2, 27.1, 26.0, 25.4, 21.3, 18.5. **HRMS** (ESI) m/z $[M+Na]^+$ calculated for $C_{15}H_{26}NaO_2^+$: 261.1825, found: 261.1827.

Synthesis of antipacid A

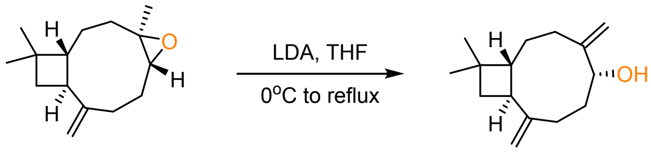

(–)-caryophyllene oxide (**1**) **9a**

(1*S*,5*R*,9*R*)-10,10-dimethyl-2,6-dimethylenebicyclo[7.2.0]undecan-5-ol (9a): In a flame dried round bottom flask flashed with argon, diisopropylamine (0.696 ml, 4.97 mmol, 1.6 equiv) was added to 6.5 ml dry THF. The mixture was cooled down to -30°C and n-BuLi (1.6 M in hexanes, 2.72 ml, 4.35 mmol, 1.4 equiv) was added dropwise. The reaction mixture was left under stirring for 1 h keeping the temperature below 0°C. Then, a solution of (-) caryophyllene oxide (**5**) (685 mg, 3.11 mmol, 1.0 equiv) in 7.1 ml THF was added dropwise giving to the mixture a pale-yellow color. The mixture was left to warm up to room temperature and then heated in an oil bath to reflux for 2 h. TLC monitoring showed completion of the reaction. The mixture was diluted with 20 ml EtOAc and quenched with aqueous *sat*. NH_4Cl (10 ml). The organic phase was separated while the aqueous one was extracted twice with EtOAc (2 x 10 ml). The combined organic phases were back washed with 20 ml NaCl 20% w/v, dried over anhydrous Na_2SO_4, and concentrated under vacuo. The oily residue was purified by silica gel flash column chromatography (cyclohexane:EtOAc = 10:1) to provide 582 mg of alcohol **9a** as a white solid (85% yield). The spectroscopic data are consistent with the reported ones.[8]

$[a]^{25}_{D}$ = +46 (*c* 0.042 M, $CHCl_3$). **^{1}H NMR** (500 MHz, $CDCl_3$) δ 5.04 (s, 1H), 4.95 (s, 1H), 4.78 (d, *J* = 1.5 Hz, 1H), 4.76 (d, *J* = 1.6 Hz, 1H), 4.09 (dd, *J* = 8.9, 3.9 Hz, 1H), 2.52 (dt, *J* = 13.6, 4.3 Hz, 1H), 2.40 – 2.25 (m, 2H), 2.05 – 1.91 (m, 2H), 1.86 – 1.69 (m, 4H), 1.67 – 1.46 (m, 3H), 0.98 (s, 6H). **^{13}C NMR** (126 MHz, $CDCl_3$) δ 152.4, 151.3, 113.5, 109.1, 75.2, 54.2, 43.8, 37.0, 33.5, 32.8, 32.6, 32.4, 30.7, 30.0, 22.0.

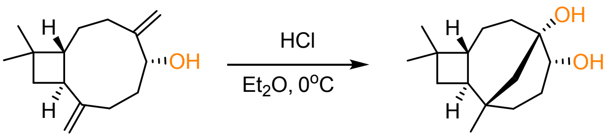

9a **16**

(1*R*,2*S*,5*R*,8*S*,9*R*)-1,4,4-trimethyltricyclo[6.3.1.0^{2,5}]dodecane-8,9-diol (16): A round bottom flask was charged with a solution of compound **9a** (495 mg, 2.25 mmol, 1.0 equiv) in Et_2O (10 ml). A solution of 2.5 ml HCl (2.0 M in Et_2O) was added dropwise under cooling at 0°C. TLC monitoring showed completion of the reaction after 2 h, at which point the reaction was

quenched with 10 ml of cold H_2O. The pH of the mixture was adjusted to neutral under cooling, using solid Na_2CO_3. Then, the organic phase was removed, while the aqueous one was extracted with EtOAc (2 x 50 ml). The combined organic phases were dried over anhydrous Na_2SO_4 and evaporated down. The oily residue was purified by silica gel flash chromatography (cyclohexane:EtOAc = 1:1) to provide 251 mg of diol **16** in 47% yield.

$[a]^{25}_D$ = +3.6 (*c* 0.5, $CHCl_3$); [lit$[a]^{25}_D$= +3 (*c* 0.5, $CHCl_3$)].[9] **^{1}H NMR** (500 MHz, $CDCl_3$) δ 3.50 (dd, *J* = 11.7, 5.4 Hz, 1H), 2.09 – 1.94 (m, 2H), 1.83 (dtd, *J* = 13.0, 5.1, 2.6 Hz, 1H), 1.79 – 1.54 (m, 4H), 1.48 (dd, *J* = 9.7, 7.8 Hz, 1H), 1.40 (dtd, *J* = 18.3, 5.9, 3.2 Hz, 2H), 1.36 – 1.21 (m, 4H), 0.99 (s, 3H), 0.98 (s, 3H), 0.80 (s, 3H).**^{13}C NMR** (126 MHz, $CDCl_3$) δ 79.2, 76.6, 48.1, 45.0, 37.7, 37.5, 35.8, 34.9, 33.3, 32.5, 30.6, 28.4, 26.0, 20.7, 20.6. **HRMS** (ESI) m/z $[M+Na]^+$ calculated for $C_{15}H_{26}NaO_2^+$: 261.1825, found: 261.1825.

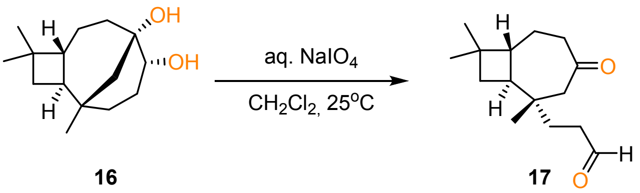

16 **17**

3-((1*S*,2*S*,7*R*)-2,8,8-trimethyl-4-oxobicyclo[5.2.0]nonan-2-yl)propanal (17): A round bottom flask was charged with a solution of diol **16** (126 mg, 0.53 mmol, 1.0 equiv) in 6.5 ml CH_2Cl_2. Then, 1.2 ml of aq. $NaIO_4$ (0.65 M, 1.5 equiv) was added to the mixture dropwise followed by 60 mg of silica. The reaction was left under stirring for 2 h, at which point TLC indicated full consumption of starting material. The reaction was diluted with 5 ml H_2O and stirred for 5 min. It was then left to settle, and organic phase was separated. The aqueous one was extracted twice with EtOAc (2 x 5 ml). The combined organic phases were dried over anhydrous Na_2SO_4 and evaporated down. The residue was purified by silica gel flash chromatography (cyclohexane:EtOAc = 5:1) to afford 108 mg of ketoaldehyde **17** as colorless oil (86% yield).

^{1}H NMR (500 MHz, $CDCl_3$) δ 9.75 (t, *J* = 1.8 Hz, 1H), 2.51 – 2.26 (m, 6H), 1.89 – 1.74 (m, 2H), 1.68 – 1.64 (m, 2H), 1.60 – 1.38 (m, 4H), 1.01 (s, 6H), 0.91 (s, 3H).**^{13}C NMR** (126 MHz, $CDCl_3$) δ 212.7, 202.4, 54.8, 46.3, 45.3, 43.9, 39.3, 35.5, 34.9, 34.4, 33.6, 30.1, 23.8, 22.1, 20.9. $[a]^{25}_D$ = -72.72 (C= 0.1 M, $CHCl_3$). **HRMS** (ESI) m/z $[M+Na]^+$ calculated for $C_{15}H_{24}NaO_2^+$: 259.1669, found: 259.1669.

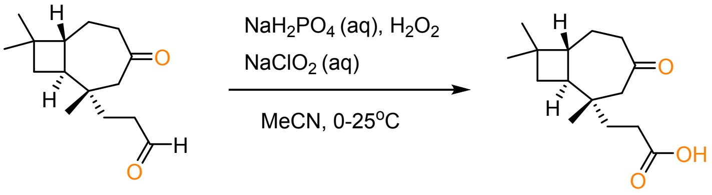

17 antipacid A (**6**)

Antipacid A (6): A round bottom flask was charged with a solution of aldehyde **17** (19.8 mg, 0.084 mmol, 1.0 equiv) in 1.0 ml CH_3CN, followed by a solution of NaH_2PO_4 (45 mg, 0.38 mmol, 4.5 equiv) in 200 µL H_2O and H_2O_2 30% w/w (0.12 ml, 0.109 mmol, 1.3 equiv) dropwise at 0ºC. The reaction mixture was stirred for 10 min at 0ºC before a solution $NaClO_2$ (15.2 mg, 0.168 mmol, 2.0 equiv) in 100 µL H_2O was added. The reaction mixture was warmed up to ambient temperature and stirred there for 2 h further at which point aldehyde was fully converted to a more polar spot as evidenced by TLC analysis. The reaction was diluted with 2.0 ml H_2O and extracted twice with EtOAc (2 x 4 ml). The organic layers were collected, dried over anhydrous Na_2SO_4 and evaporated down to dryness. The residue was purified by column chromatography using cyclohexane:EtOAc = 1:2 as the eluent. Antipacid A (16.3 mg, 0.066 mmol) was collected as a colorless thick oil in 78% yield.

$[a]^{25}_{D}$ = -45 (C= 0.016 M, $CHCl_3$); [lit$[a]^{25}_{D}$= -9.2 (*c* 0.29, $CHCl_3$)].[10] **^{1}H NMR** (500 MHz, $CDCl_3$) δ 2.50 (m, 1H), 2.43 (m, 1H), 2.34 (m, 1H), 2.32 (d, 1H)*, 2.25 (m, 1H), 1.85 (ddd, J= 10.4, 10.4, 8.0 Hz, 1H), 1.72 (ddd, J= 10.4, 10.4, 3.6 Hz, 1H), 1.69 (m, 2H), 1.59 -1.60 (m, 2H), 1.49- 1.52 (m, 2H), 1.01 (s, 6H), 0.91 (s, 3H) **^{13}C NMR** (126 MHz, $CDCl_3$) δ 212.9, 179.2, 54.7, 46.4, 45.2, 43.8, 36.9, 35.5, 35.1, 34.3, 30.1, 29.4, 23.8, 22.2, 20.7. **HRMS** (ESI) m/z $[M+H]^+$ calculated for $C_{15}H_{25}O_3^+$: 253.1798, found: 253.1798.

References

1. Vogel, A. I. Vogel's textbook of practical organic chemistry; Longman Scientific & Technical, 1989.
2. Cai, L. Current Protocols Essential Laboratory Techniques 2014, 8 (1).
3. Bhunia, A.; Bergander, K.; Daniliuc, C. G.; Studer A. Fe-Catalyzed Anaerobic Mukaiyama-Type Hydration of Alkenes using Nitroarenes. *Angew. Chem. Int. Ed.* **2021**, *60*, 8313–8320.
4. Tkachev, A. V.; Dubovenko, Zh. V.; Pentegova, V. A. *J. Org. Chem.* USSR (English Translation), **1985**, *21*, 1593−1601.
5. Bombarda, I.; Gaydou, E. M.; Smadja, J.; Faure, R. Synthèse de composés oxygénés dérivés du caryophyllene. *Bull. Soc. Chim. Fr.* **1995**, *132*, 836–842.
6. Ranieri, B.; Obradors, C.; Mato, M.; Echavarren, A. M. Synthesis of Rumphellaone A and Hushinone by a Gold-Catalyzed [2 + 2] Cycloaddition. *Org. Lett.* **2016***, 18,* 1614–1617.
7. Chung, H. M.; Wang, W. H.; Hwang, T. L.; Wu, Y. C.; Sung, P. J. Natural Caryophyllane Sesquiterpenoids from Rumphella antipathies. *Nat Prod Commun.* **2015**, *10*, 835–838.
8. Stakanovs, G.; Belyakov, S.; Jirgensons, a.; Rasina, D. Convergent biomimetic semisynthesis of disesquiterpenoid rumphellolide J. *Org. Biomol. Chem.* **2022**, *20*, 2455–2461.
9. Racero, J. C.; Macías-Sánchez, A. J.; Hernández-Galán, R.; Hitchcock, P. B.; Hanson, J. R.; Collado I. G. Novel Rearrangement of an Isocaryolane Sesquiterpenoid under Mitsunobu Conditions *J. Org. Chem.* **2000**, *65*, 7786–7791.
10. Chang, Y.-C.; Chiang, C.-C.; Chang, Y.-S.; Chen, J.-J.; Wang, W.-H.; Fang, L.-S.; Chung, H.-M.; Hwang, T.-L.; Sung, P.-J. Novel Caryophyllane-Related Sesquiterpenoids with Anti-Inflammatory Activity from Rumphella antipathes (Linnaeus, 1758) *Mar. Drugs* **2020**, *18*, 554−563.

Copies of NMR spectra

Rumphellolide K

(*1R*,*4R*,*6R*,*9S*,*10S*)-4,9,12,12-Tetramethyl-5-oxatricyclo[8.2.0.0^{4,6}]dodecan-9-ol (10)

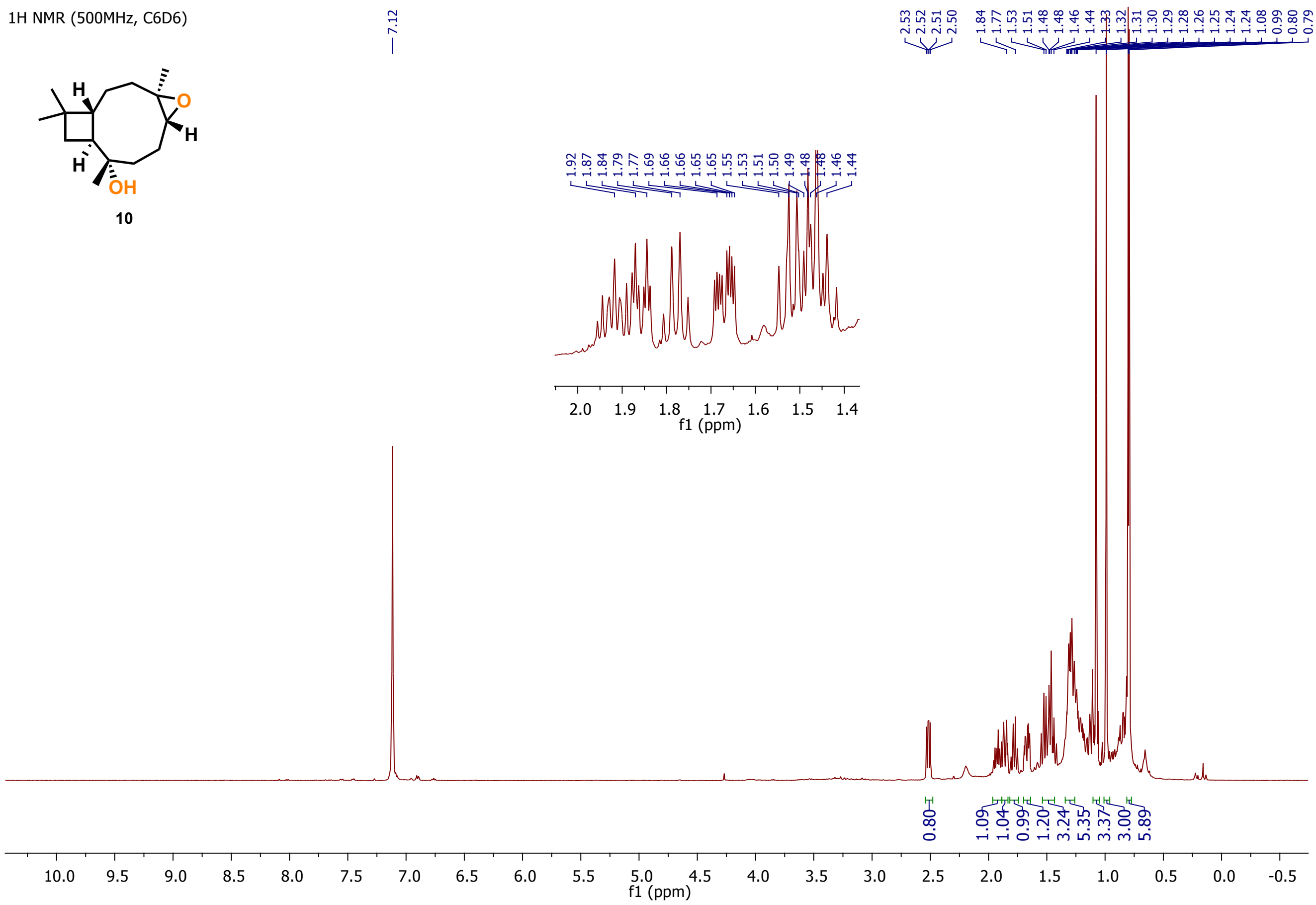

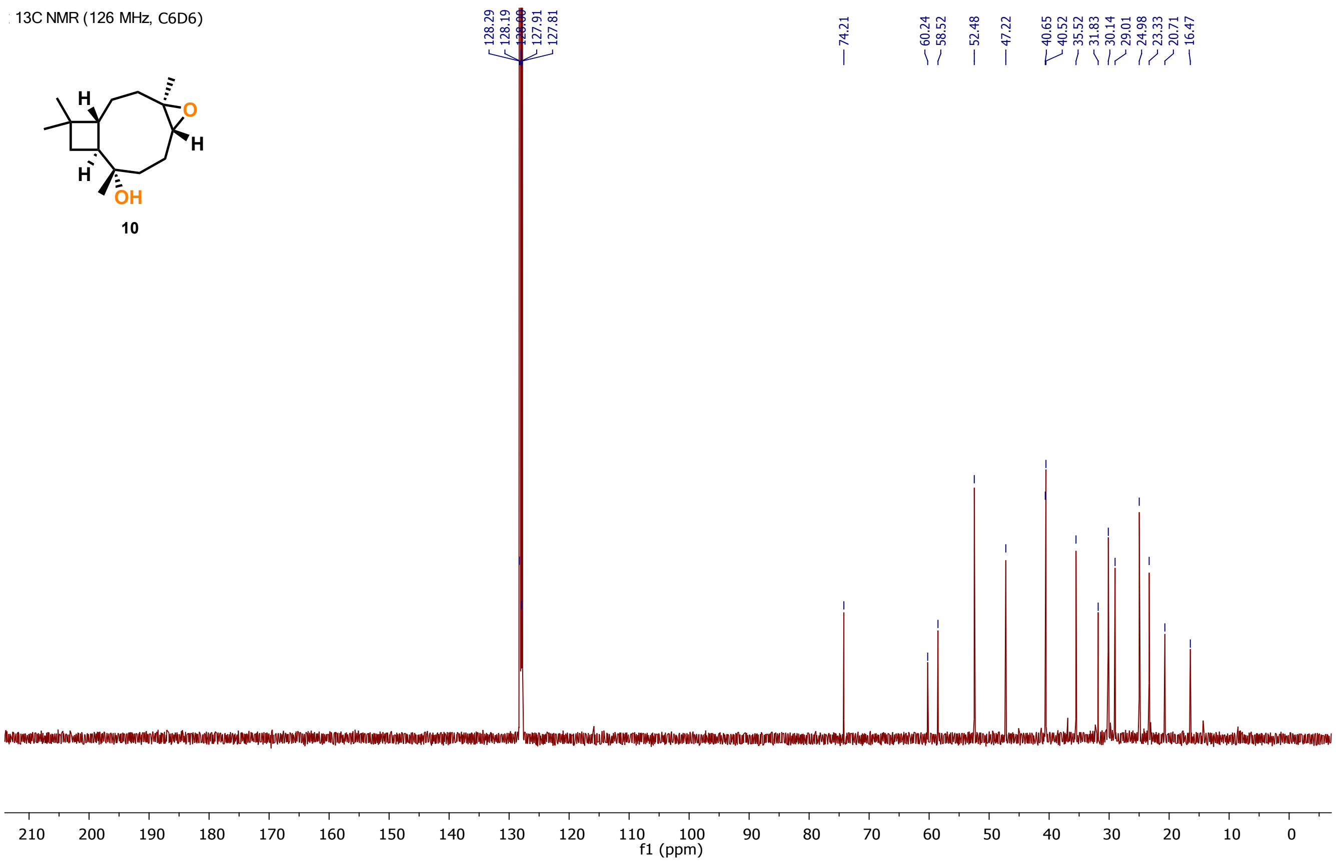

(1*S*,2*S*,5*R*,9*R*,*Z*)-2,6,10,10-tetramethylbicyclo [7.2.0]undec-6-ene-2,5-diol (8)

1H NMR (500MHz, CDCL3)

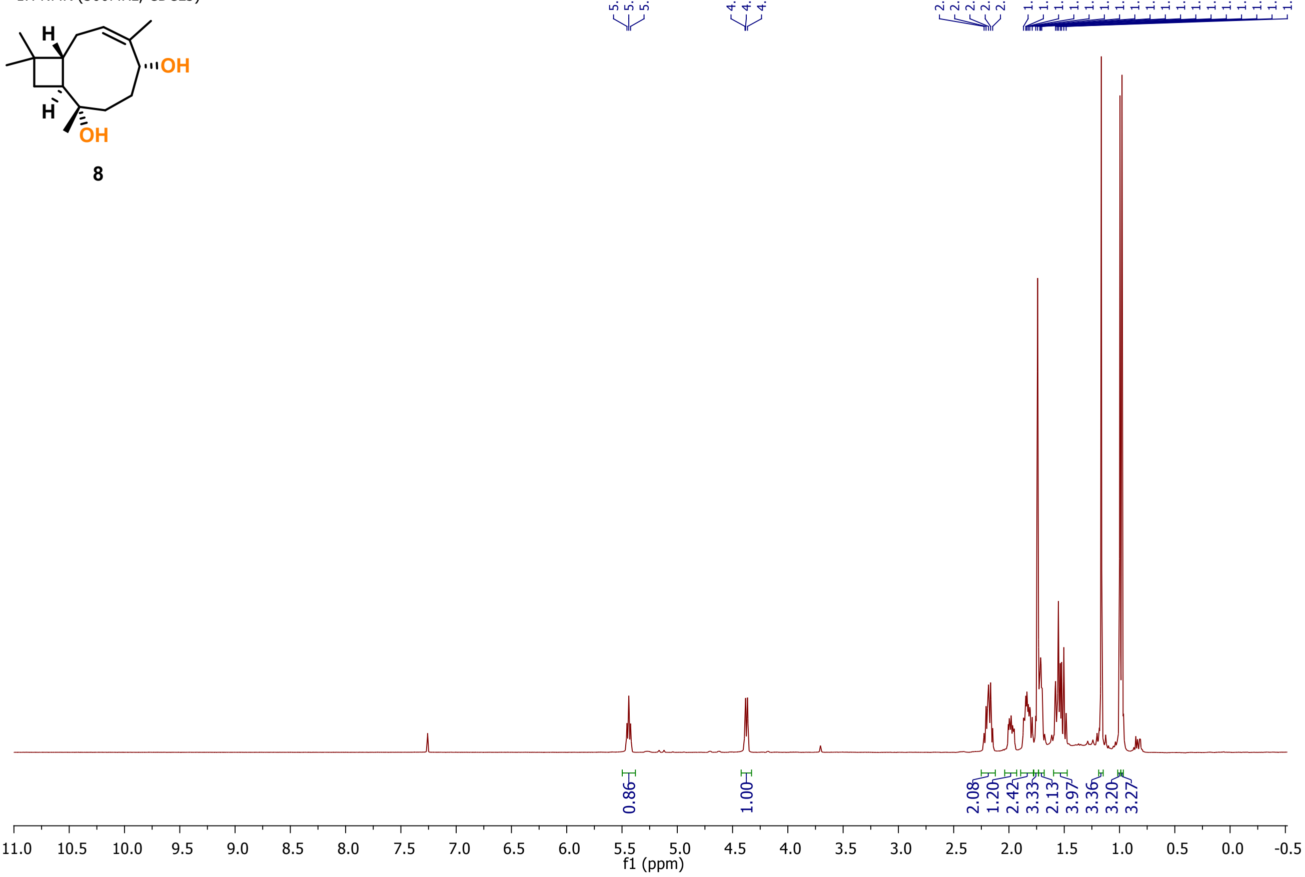

13C NMR (126 MHz, CDCL3)

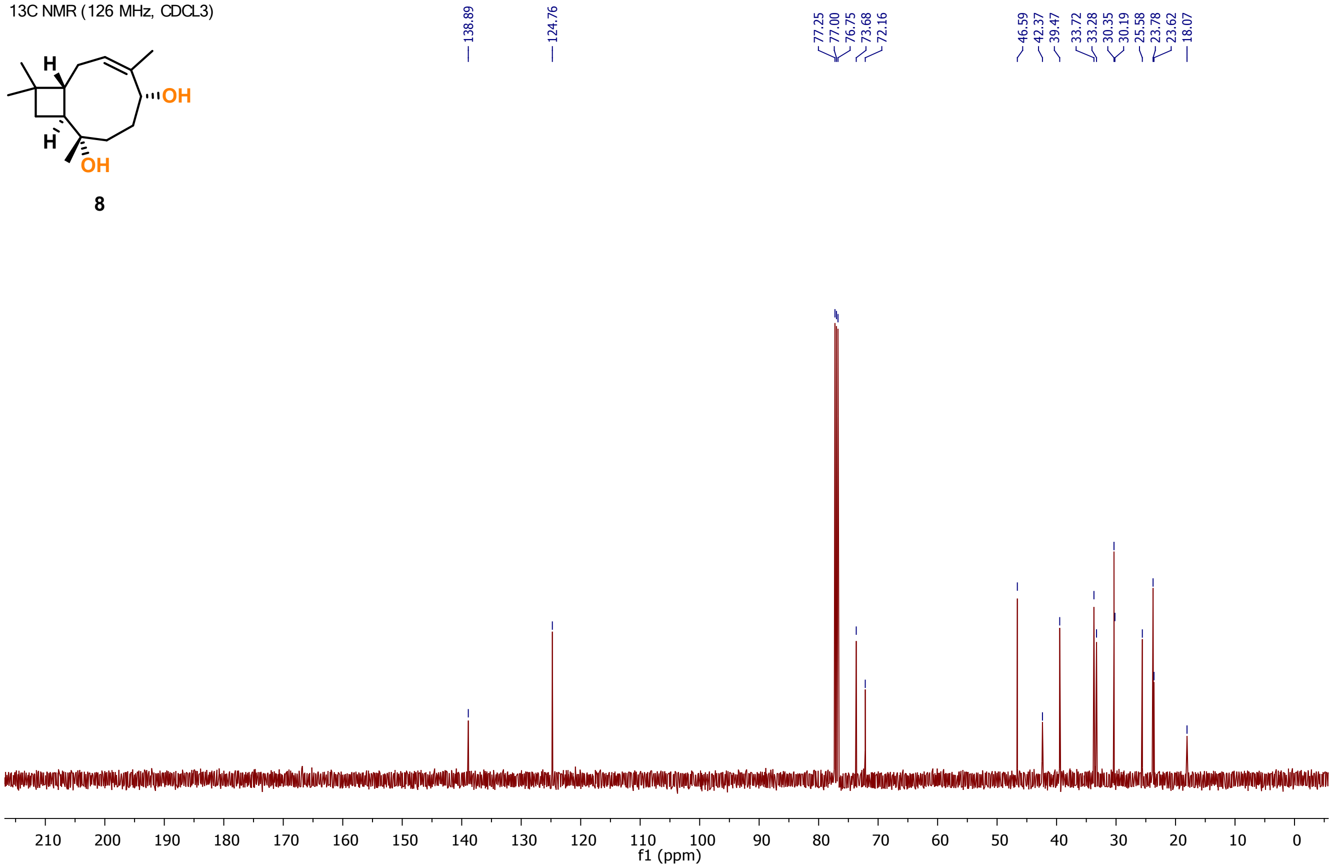

(1*R*,3*R*,5*S*,6*R*,9*S*,10*S*)-5,9,12,12-tetramethyl-4-oxatricyclo[8.2.0.0^{3,5}]dodecane-6,9-diol (7)

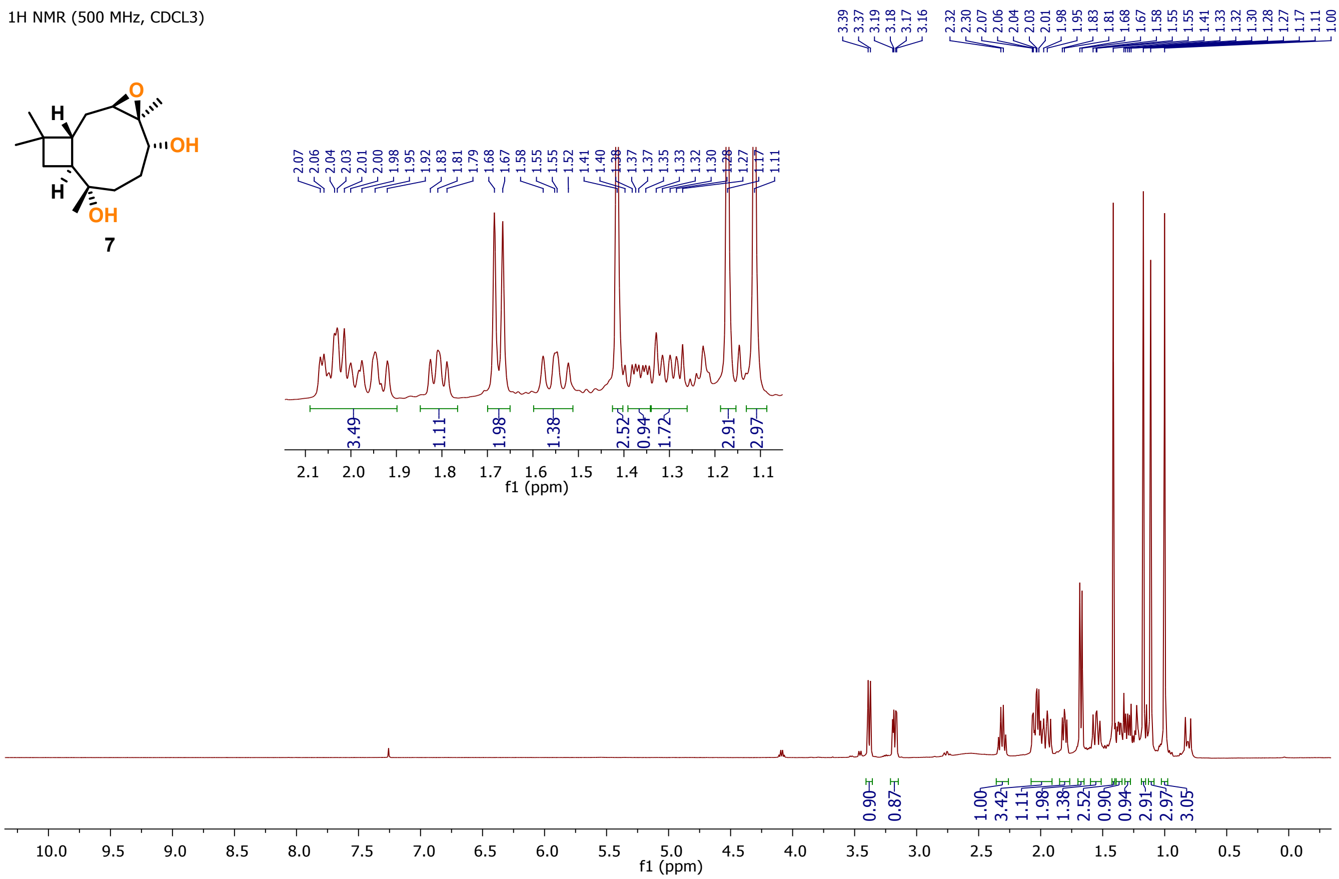

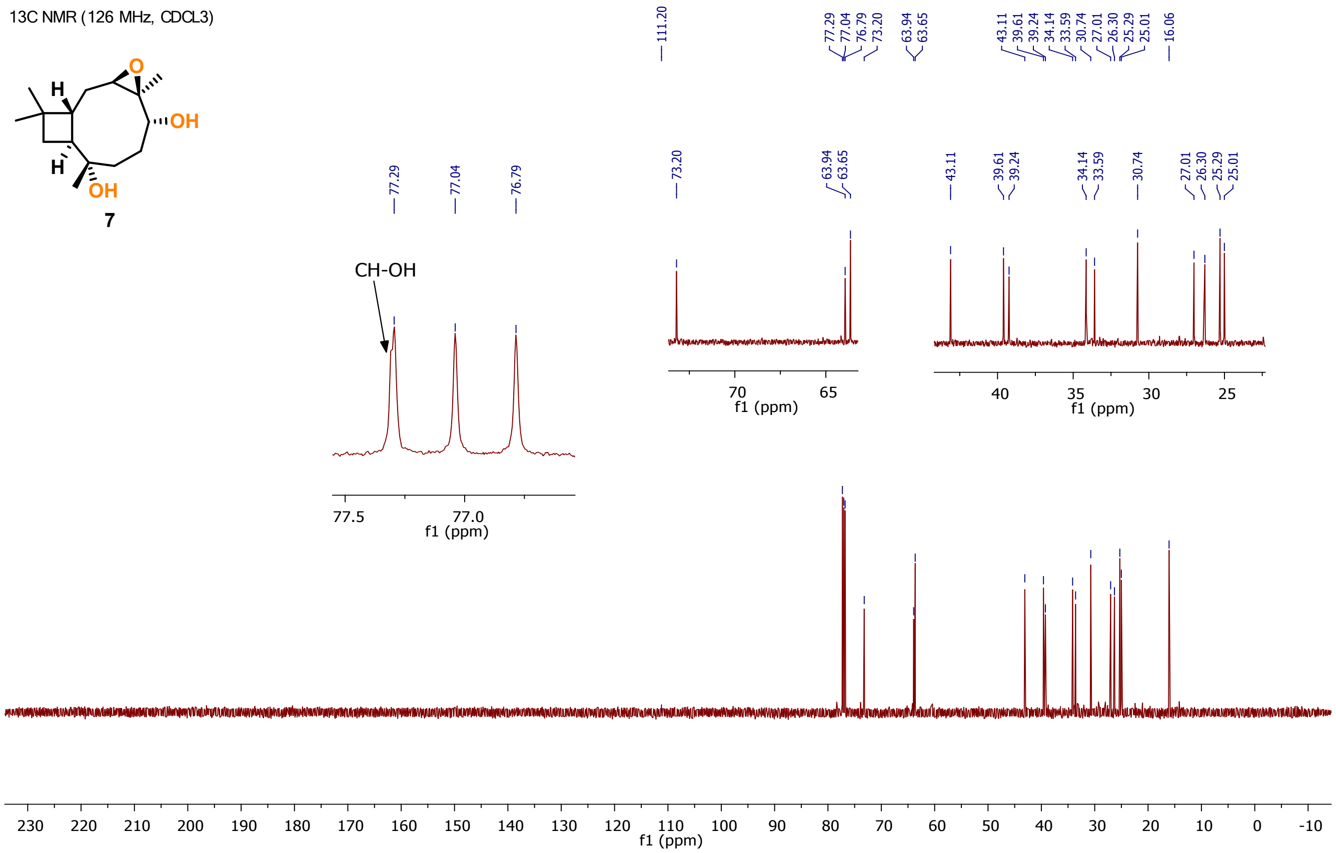

Rumphellolide K (4)

1H NMR (500 MHz, CDCL3)

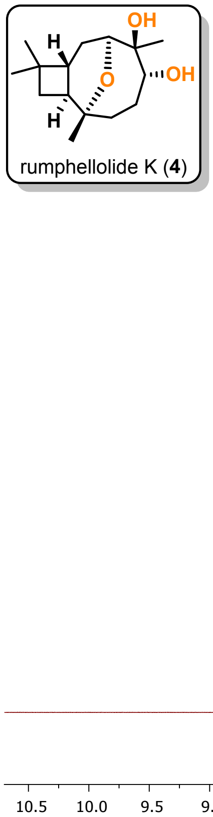

rumphellolide K (**4**)

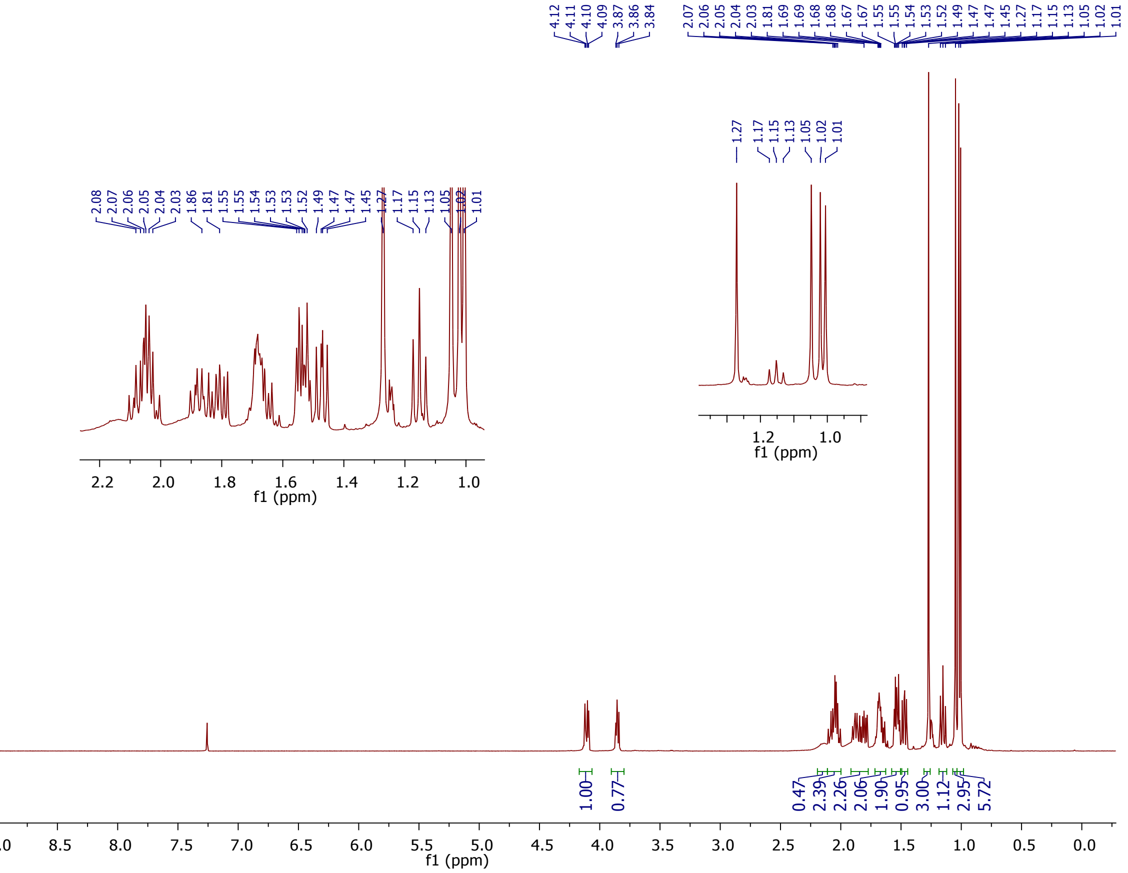

13C NMR (126 MHz, CDCL3)

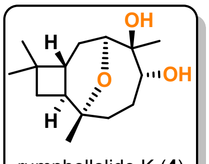

rumphellolide K (**4**)

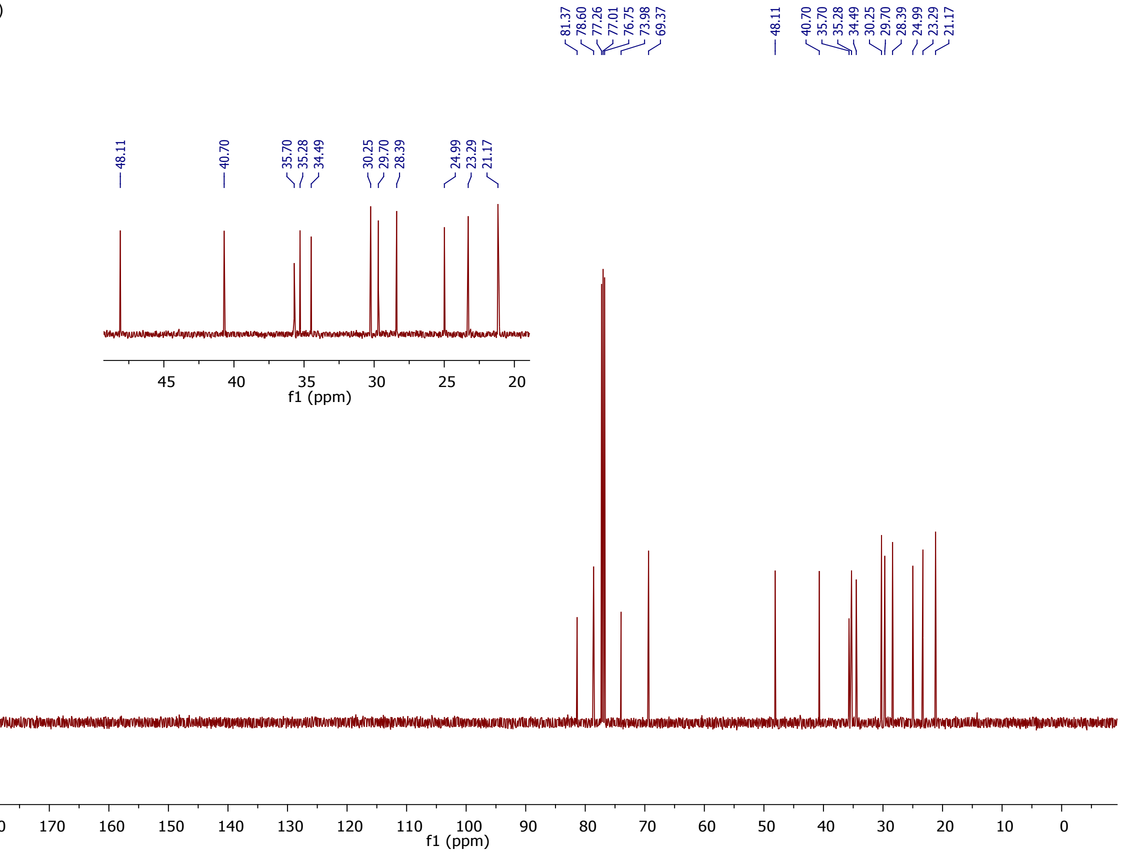

Table S3: Comparison of NMR data of synthetic and isolated rumphellolide K

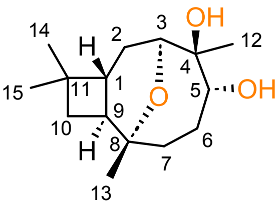

rumphellolide K (**4**)

**Position**	**This work**<br>**δ$_H$, type (*J* in Hz)**	**Isolated***<br>**δ$_H$, type, (*J* in Hz)**	**This work**<br>**δ$_C$**	**Isolated***<br>**δ$_C$, type**
**1**	2.11- 2.00, m	2.05, m	40.7	40.7, CH
**2/2'**	2.11- 2.00, m/ 1.57-1.50, m	2.06, m/ 1.53, m	34.5	34.6, CH_2
**3**	3.88- 3.83, m	3.86, dd (7.6, 1.2)	78.6	78.6, CH
**4**			81.4	81.4, C
**5**	4.11, dd (10.6, 4.5)	4.12, dd (10.0, 4.8)	69.4	69.4, CH
**6**	1.74-1.67, m/ 1.68-1.60, m	1.69, m	25.0	25.0, CH_2
**7/7'**	1.85-1.77, m/ 1.57-1.50, m	1.82, m/ 1.56, m	28.4	28.4, CH_2
**8**			74.0	74.0, C
**9**	1.92- 1.85, m	1.88, ddd (10.8, 10.8, 8.0)	48.1	48.2, CH
**10/10'**	1.47, dd (10.1, 7.7)/ 1.15, t (10.5)	1.48, dd (10.0, 8.0)/ 1.16, dd (10.8, 10.0)	35.3	35.3, CH_2
**11**			35.7	35.7, C
**12**	1.27, s	1.28, s	23.3	23.3, Me
**13**	1.05, s	1.06, s	29.7	29.7, Me
**14**	1.02, s	1.03, s	30.3	30.2, Me
**15**	1.01, s	1.01, s	21.2	21.2, Me

*From *Rumphella antipathies (Heterocycles*, Vol 100, No 9, 2020)

(1*S*,2*S*,5*R*,8*S*,9*R*)-1,4,4,8-tetramethyl-12-oxatricyclo[6.3.1.0^{2,5}]dodecan-9-ol (11)

1H NMR (500 MHz, CDCL3)

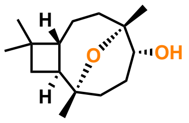

11

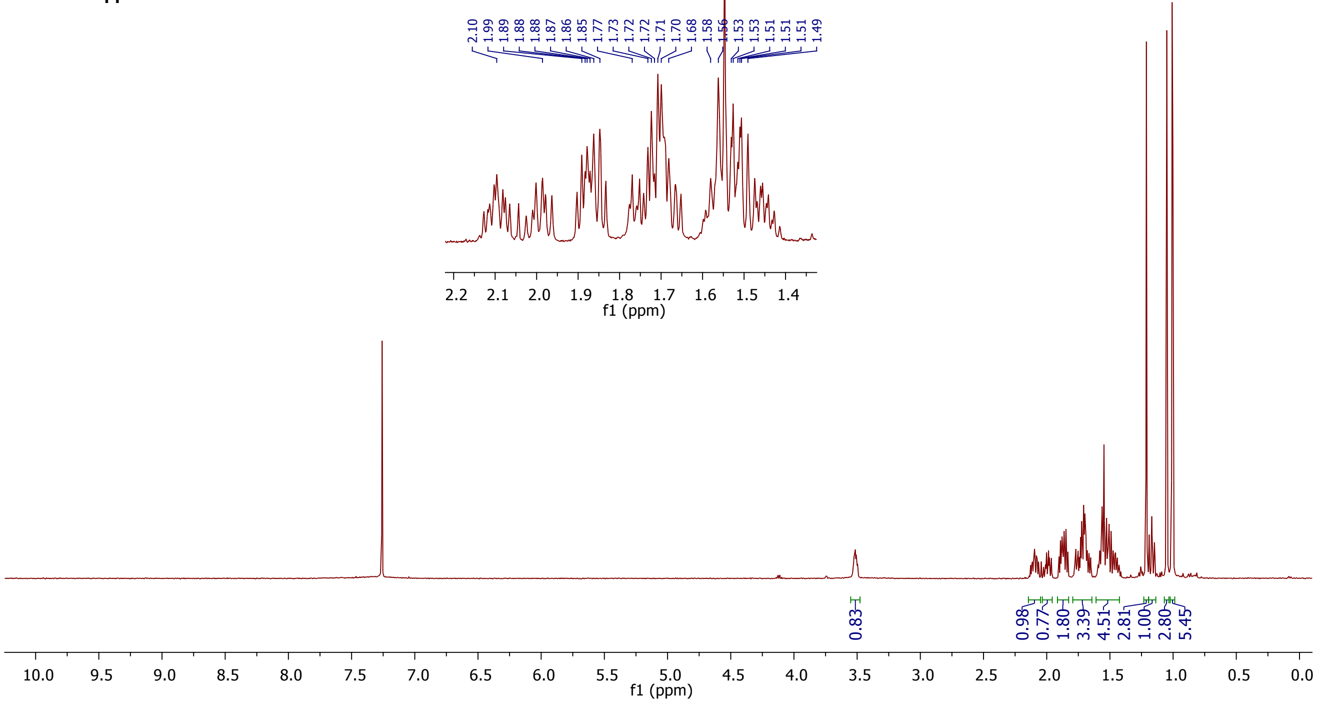

13C NMR (126 MHz, CDCL3)

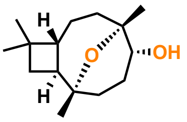

11

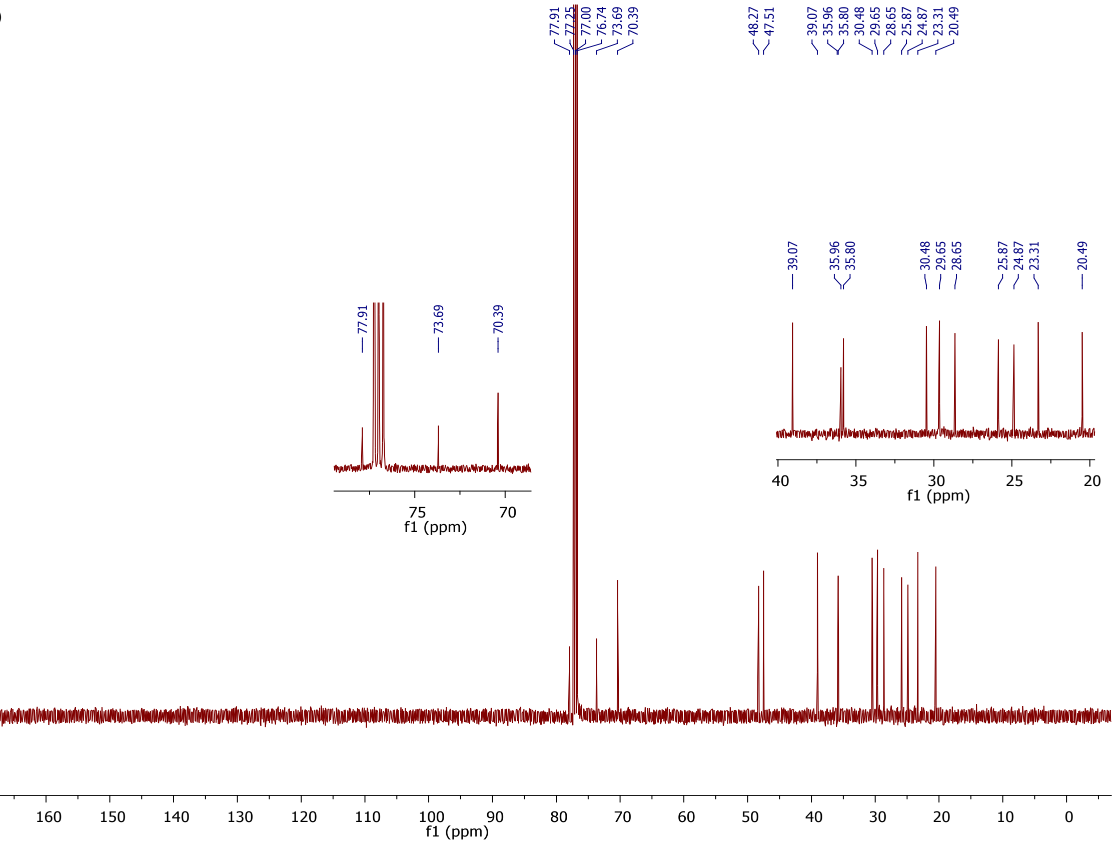

(1*S*,2*S*,5*R*,7*R*,8*R*,9*R*)-1,4,4,8-tetramethyl-12-oxatricyclo[6.3.1.0^{2,5}]dodecane-7,9-diol (12)

1H NMR (500 MHz, CDCL3)

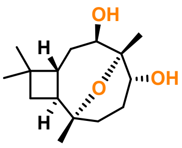

12

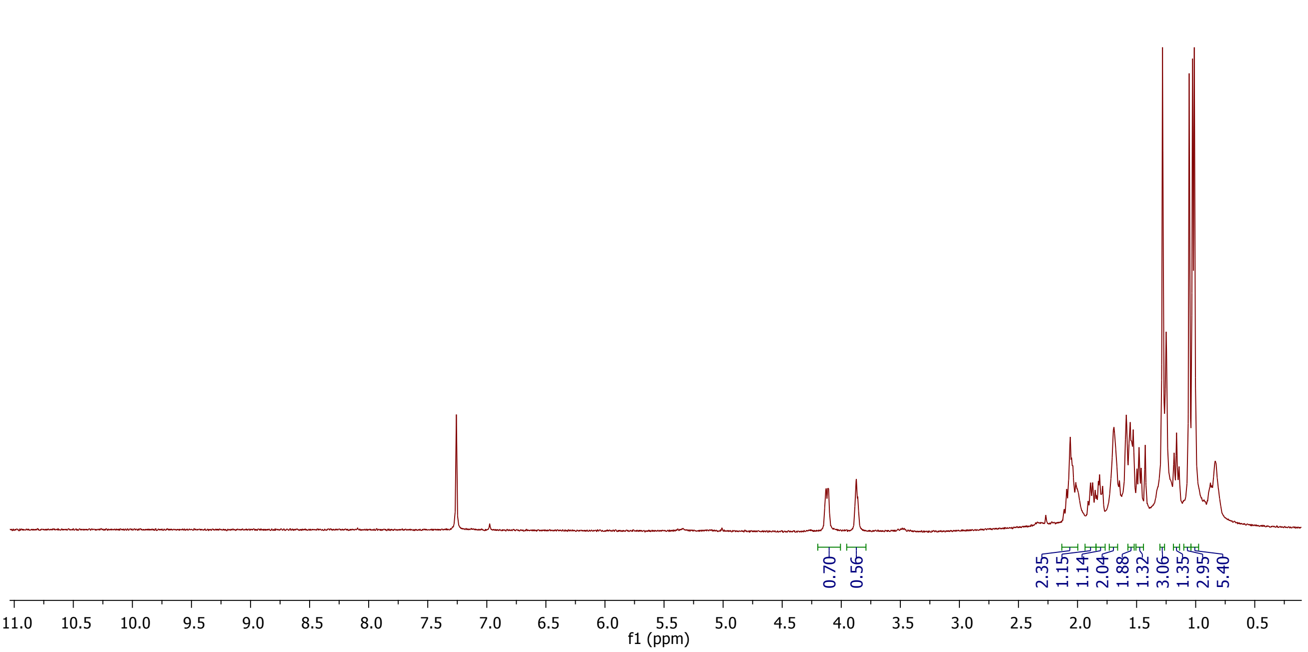

13C NMR (126 MHz, CDCL3)

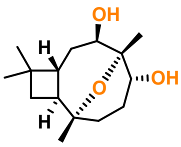

12

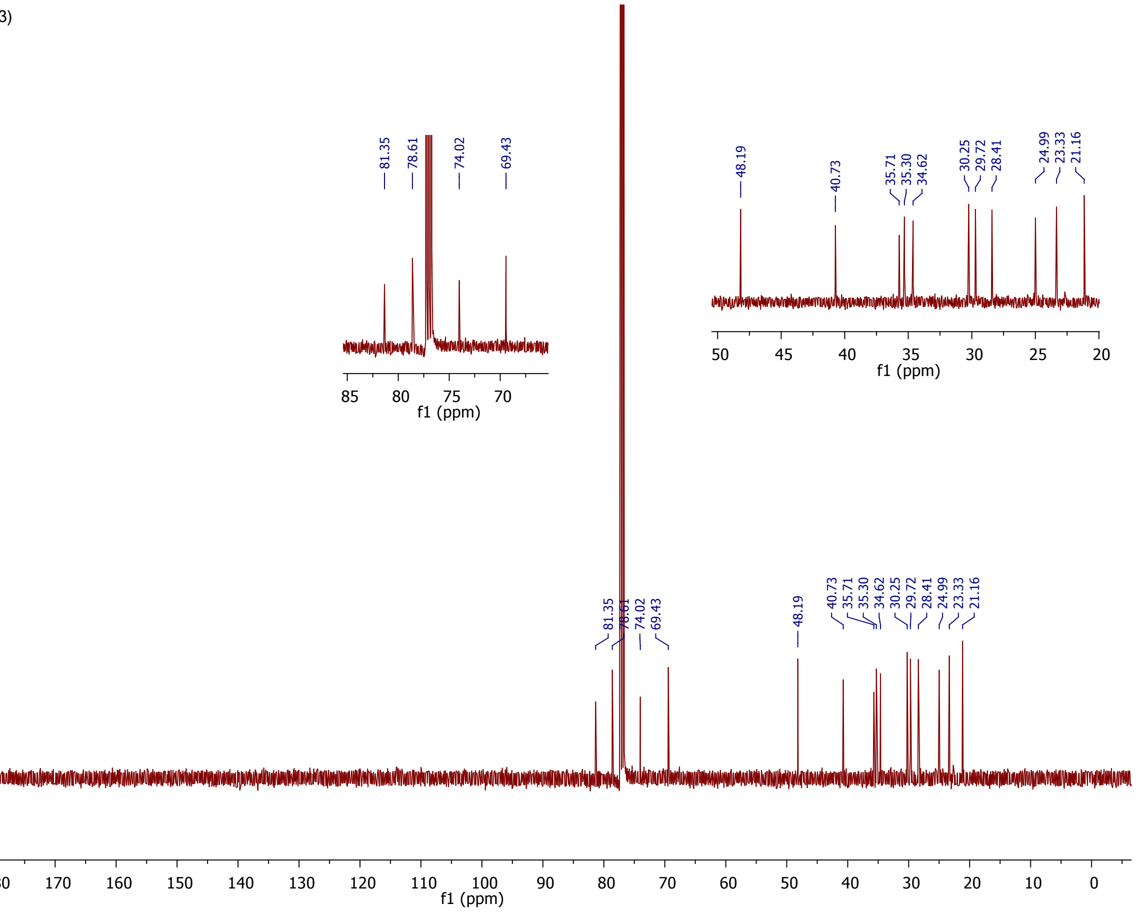

Rumphellaone A

(1*S*,2*S*,5*R*,6*R*,9*R*)-2,6,10,10-tetramethylbicyclo[7.2.0]undecane-2,5,6-triol (13)

1H NMR (500 MHz, CDCL3)

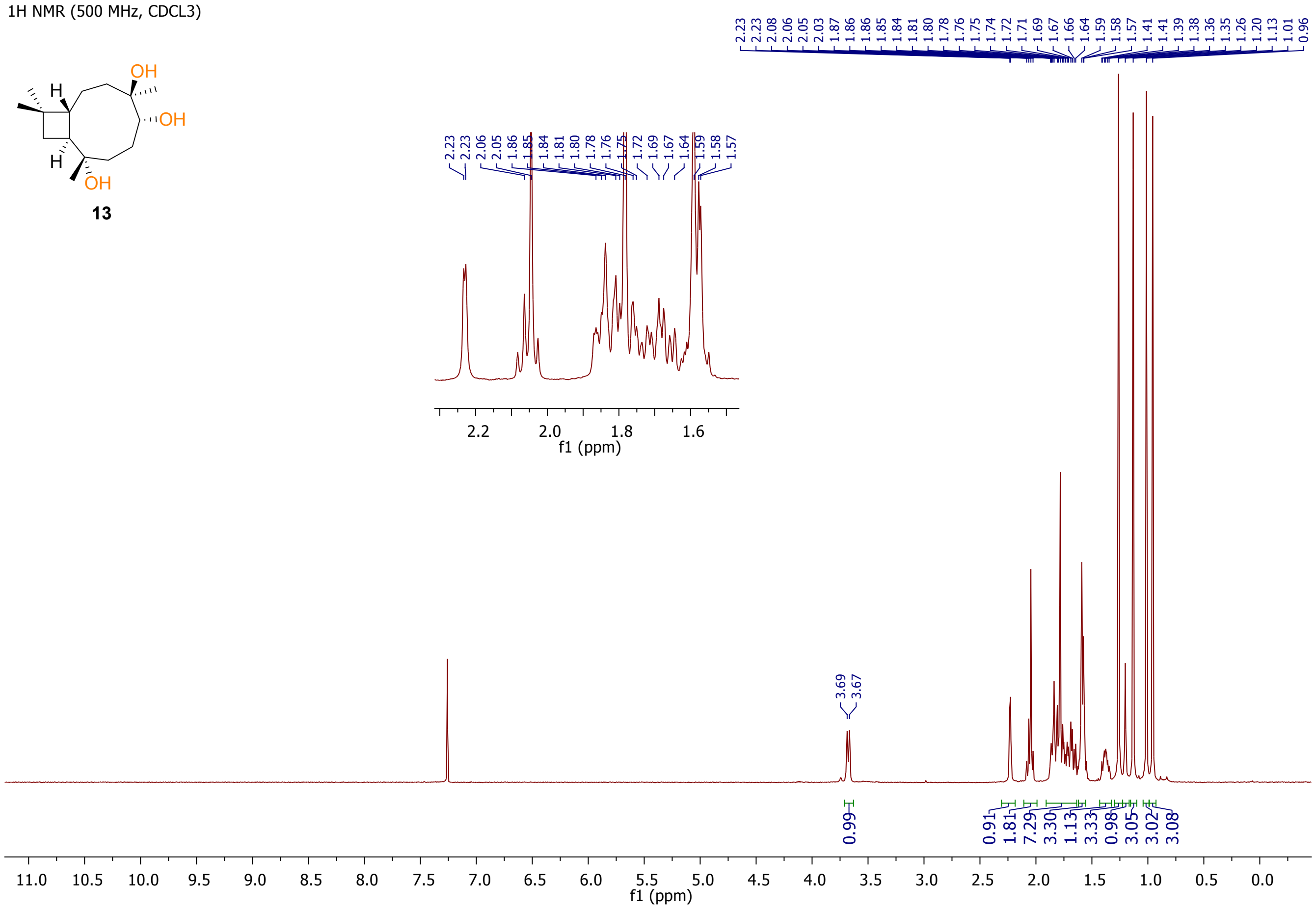

13C NMR (126 MHz, CDCL3)

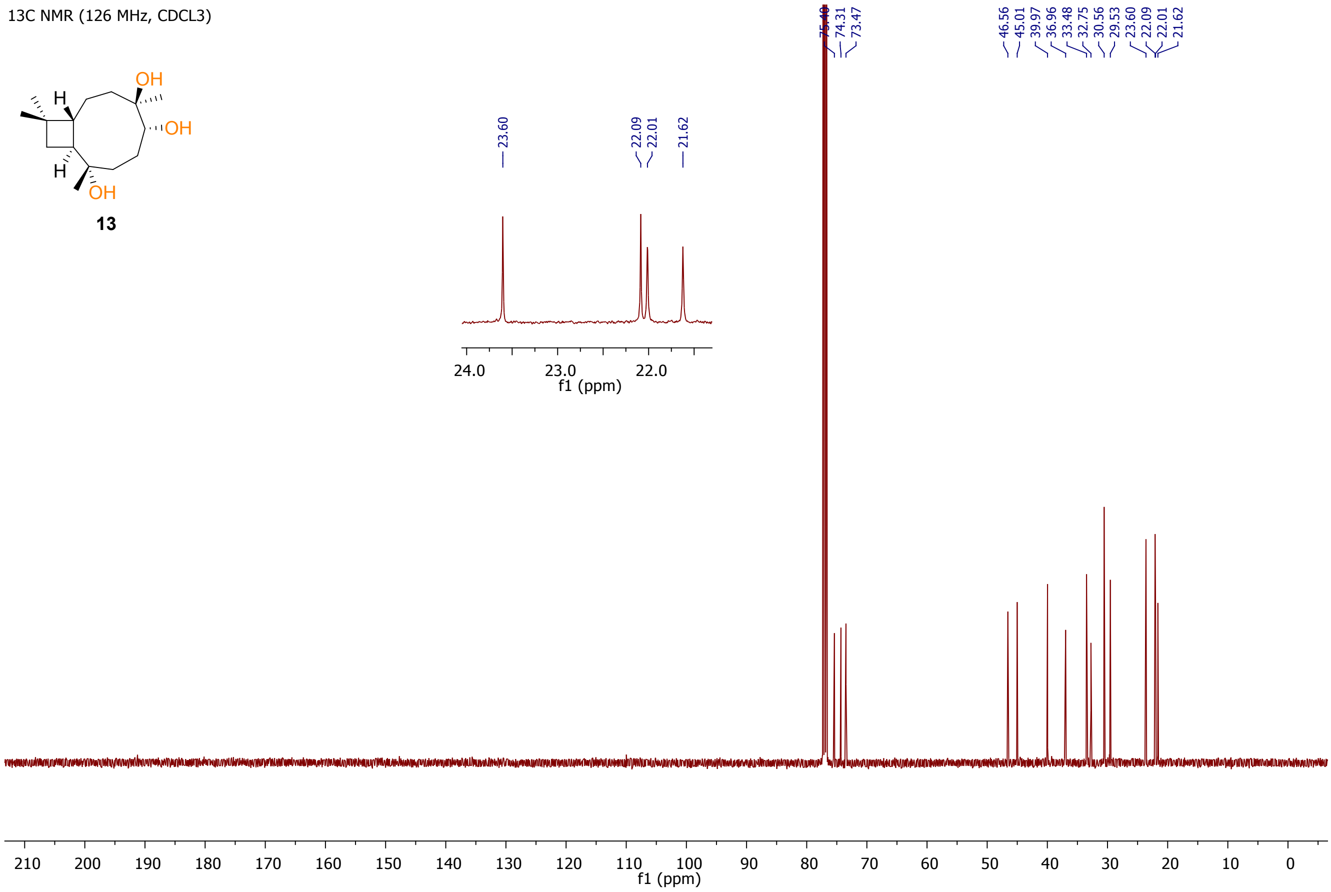

4-((1*R*,4*S*)-4-((2*S*)-5-hydroxy-2-methyltetrahydrofuran-2-yl)-2,2-dimethylcyclobutyl)butan-2-one (14)
(1:1 mixture of anomers)

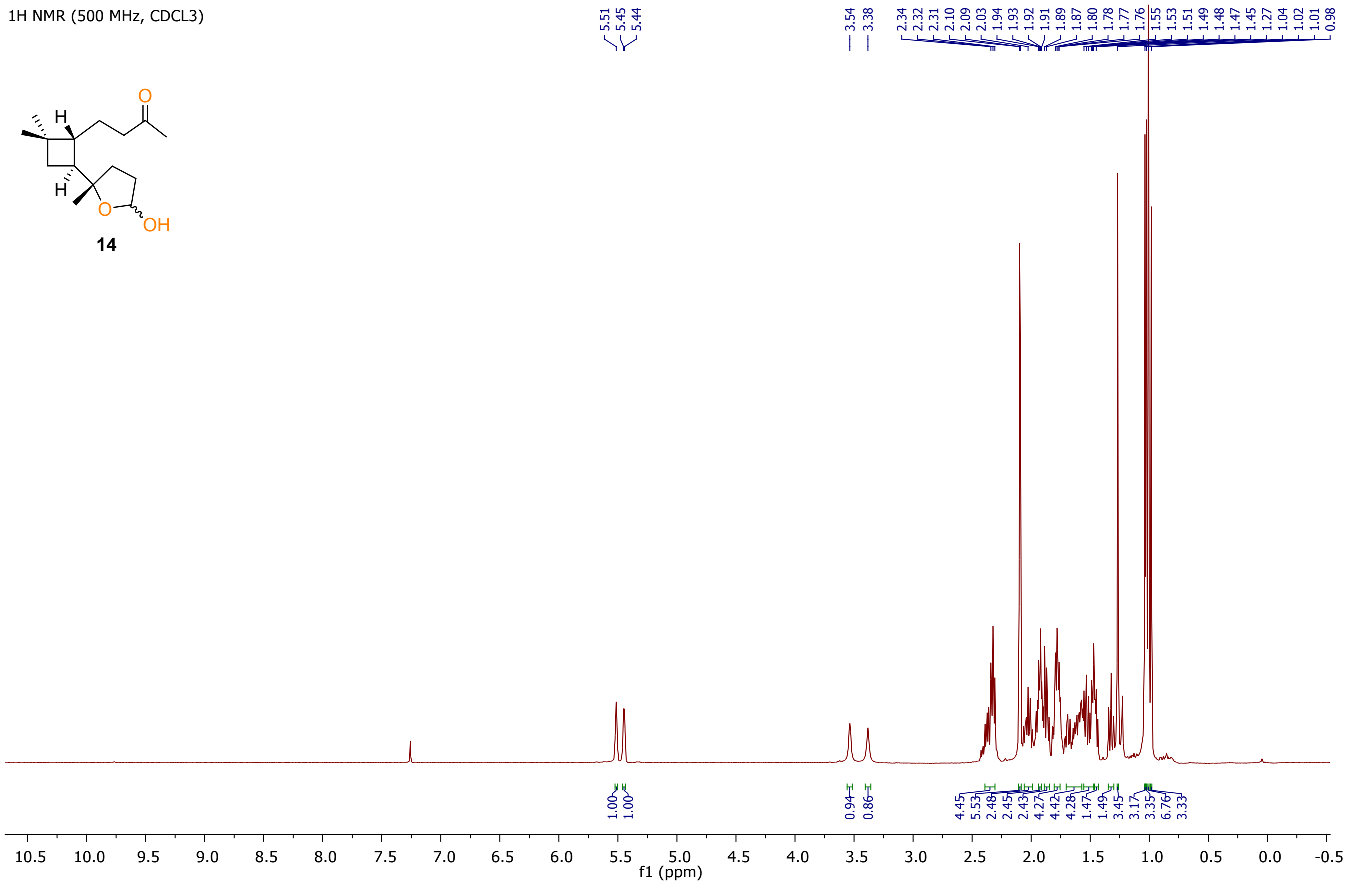

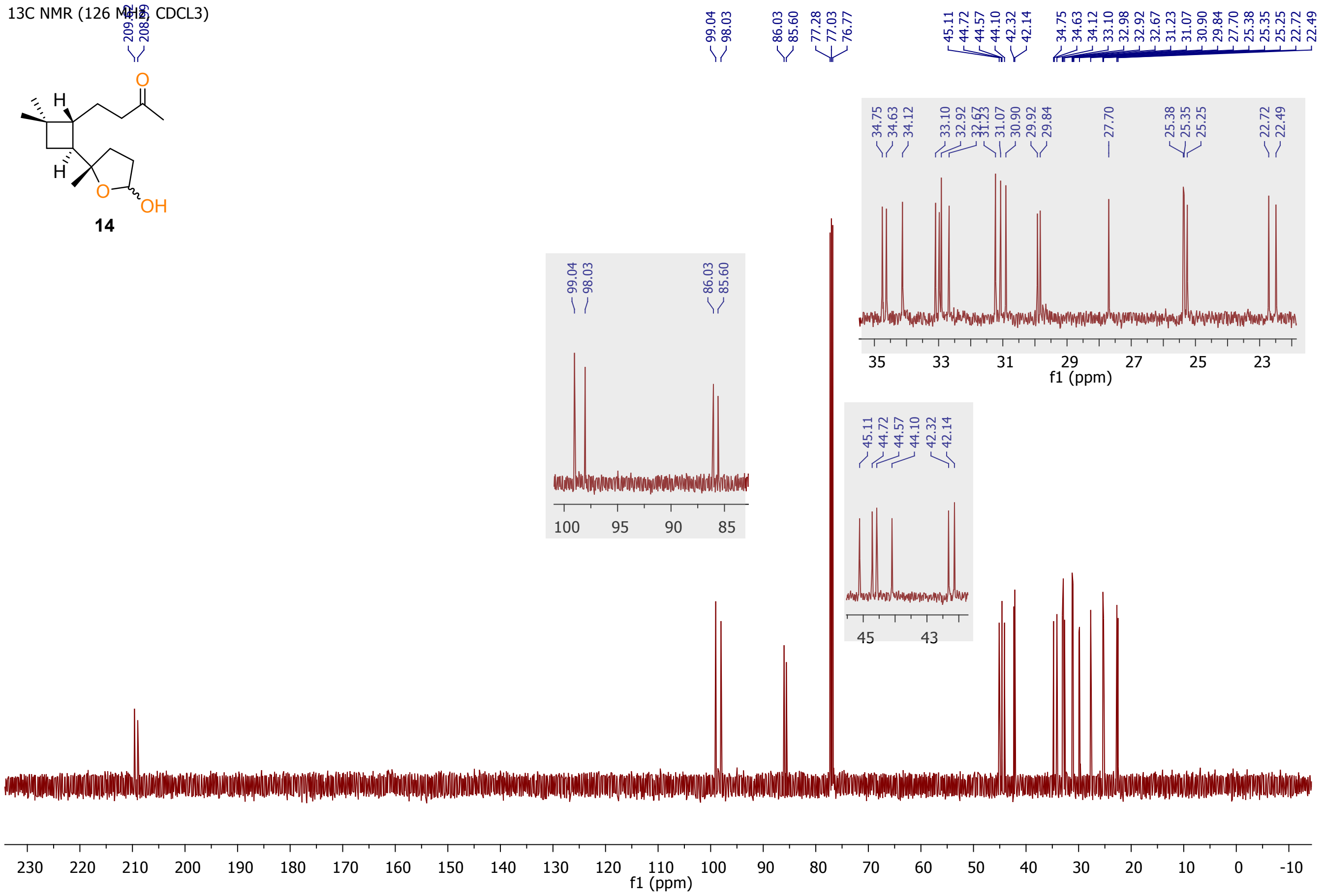

Rumphellaone A (5)

1H NMR (500 MHz, CDCL3)

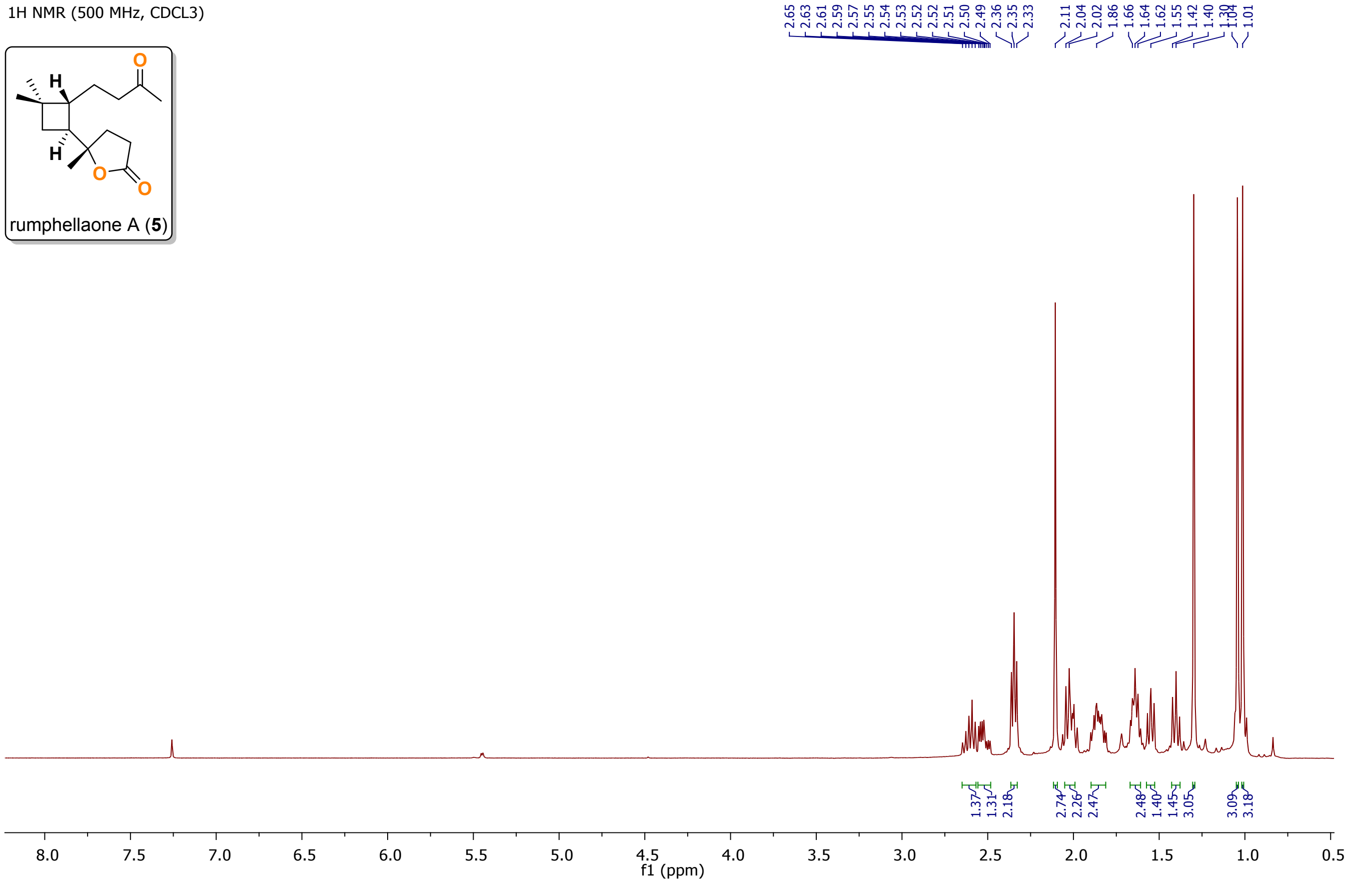

13C NMR (126 MHz, CDCL3)

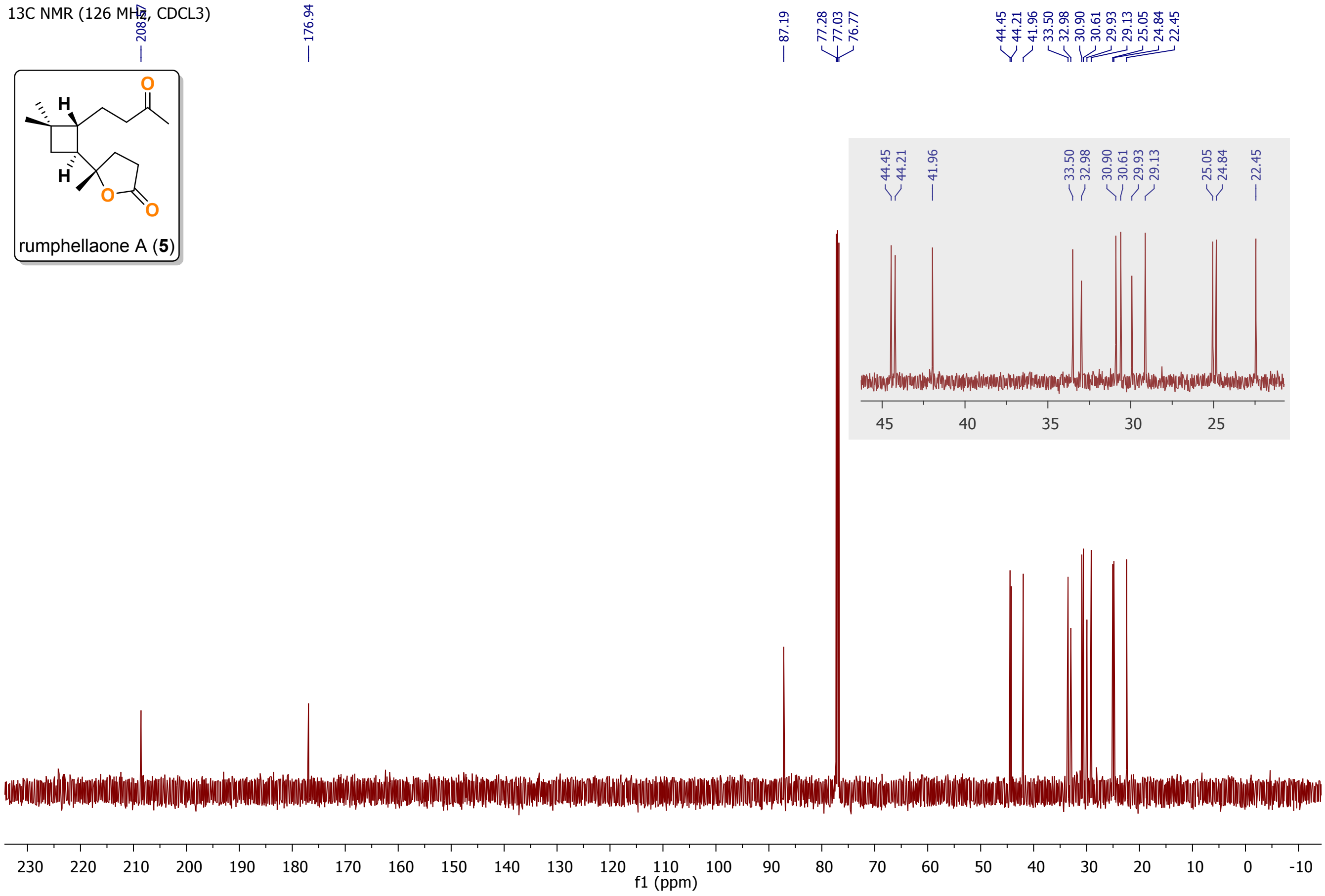

Table S4: Comparison of NMR data of synthetic and isolated rumphellaone A

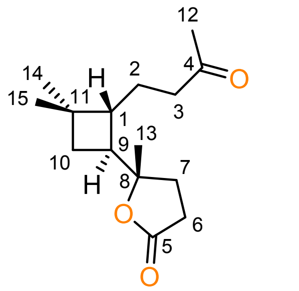

rumphellaone A (**5**)

**Position**	**This work**	**Isolated‡**	**This work**	**Isolated‡**
	**δ_H, type (*J* in Hz)**	**δ_H, type (*J* in Hz)**	**δ_C**	**δ_C, type**
**1**	1.90 – 1.81 m*	1.91 ddd (10.0, 9.2, 5.6)	44.5	44.5, CH
**2**	1.67 – 1.61 m	1.67 m	25.1	25.1, CH_2
**3**	2.35 t (7.7)	2.37 t (8.0)	42.0	42.0, CH_2
**4**	-	-	208.6	208.6, C
**5**	-	-	176.9	177.0, C
**6α/6β**	2.65 – 2.55 m/2.52 ddd (18.1, 10.0, 4.9)	2.63 ddd (18.0, 9.6, 8.8)/ 2.54 ddd (18.0, 10.0, 4.8)	29.1	29.2, CH_2
**7α/7β**	1.90–1.81 m*/2.07–1.98 m*	1.84 m/2.01 m	30.6	30.6, CH_2
**8**	-	-	87.2	87.2, C
**9**	2.07 – 1.98 m*	2.06 ddd (10.4, 10.0, 10.0)	44.2	44.3, CH
**10α/10β**	1.55 t (9.9)/1.41, t (10.4)	1.57 dd (10.0, 10.0) / 1.42 dd (10.4, 10.0)	33.5	33.6, CH_2
**11**	-	-	33.0	33.0, C
**12**	2.11, s	2.13 s	29.9	29.9, CH_3
**13**	1.30, s	1.31 s	24.8	24.9, CH_3
**14**	1.01, s	1.03 s	22.5	22.5, CH_3
**15**	1.04, s	1.07 s	30.9	30.9, CH_3

* Proton signal partly overlaps with another proton signal
‡ From *Rumphella antipathies*, Tetrahedron Lett. 2010, 51, 6025–6027

4β,8β-Epoxycaryophyllan-5-ol (15)

1H NMR (500 MHz, CDCL3)

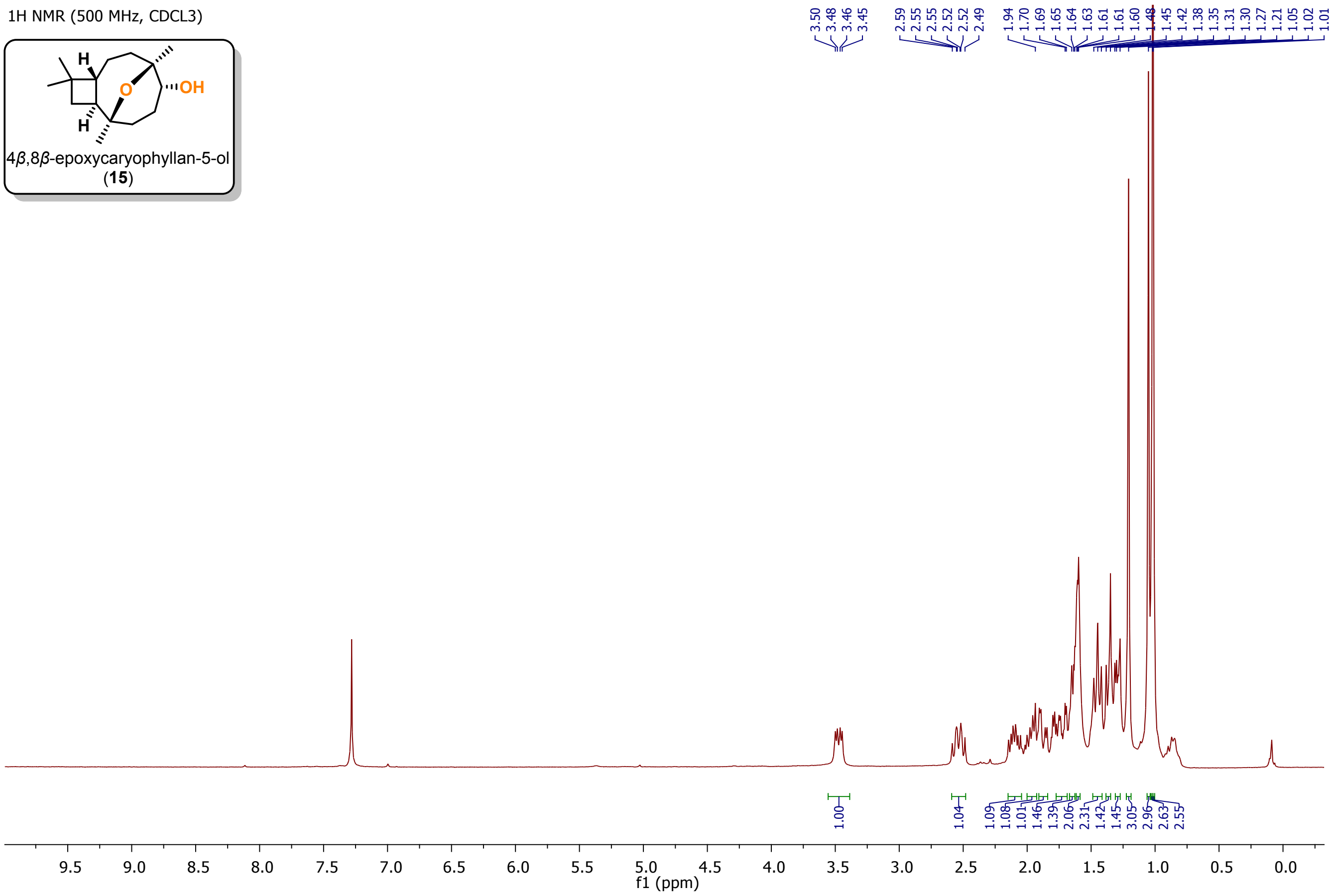

13C NMR (126 MHz, CDCL3)

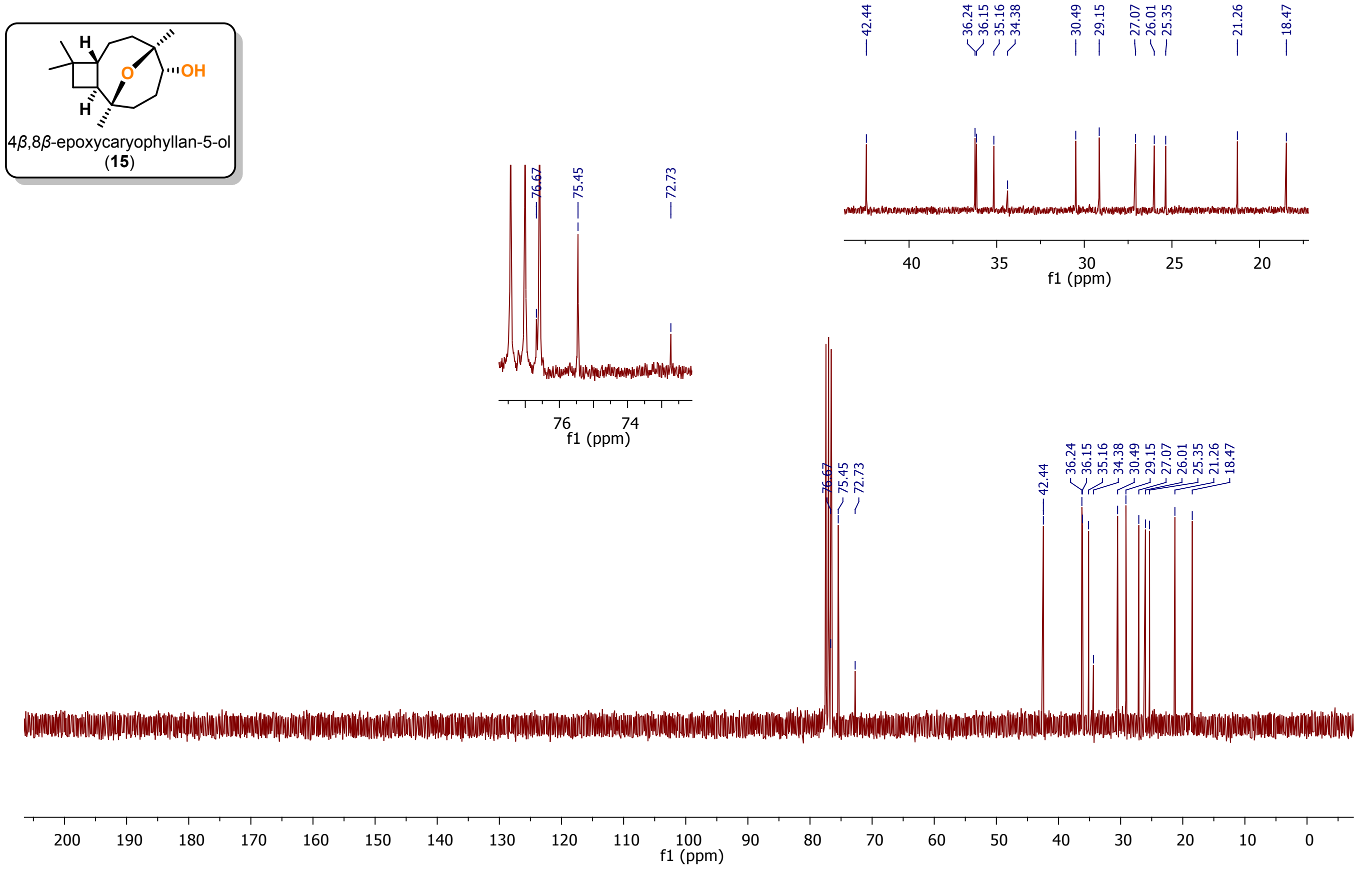

Antipacid A

(1*S*,5*R*,9*R*)-10,10-dimethyl-2,6-dimethylenebicyclo[7.2.0]undecan-5-ol (9a)

1H NMR (500 MHz, CDCL3)

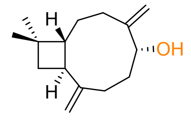

9a

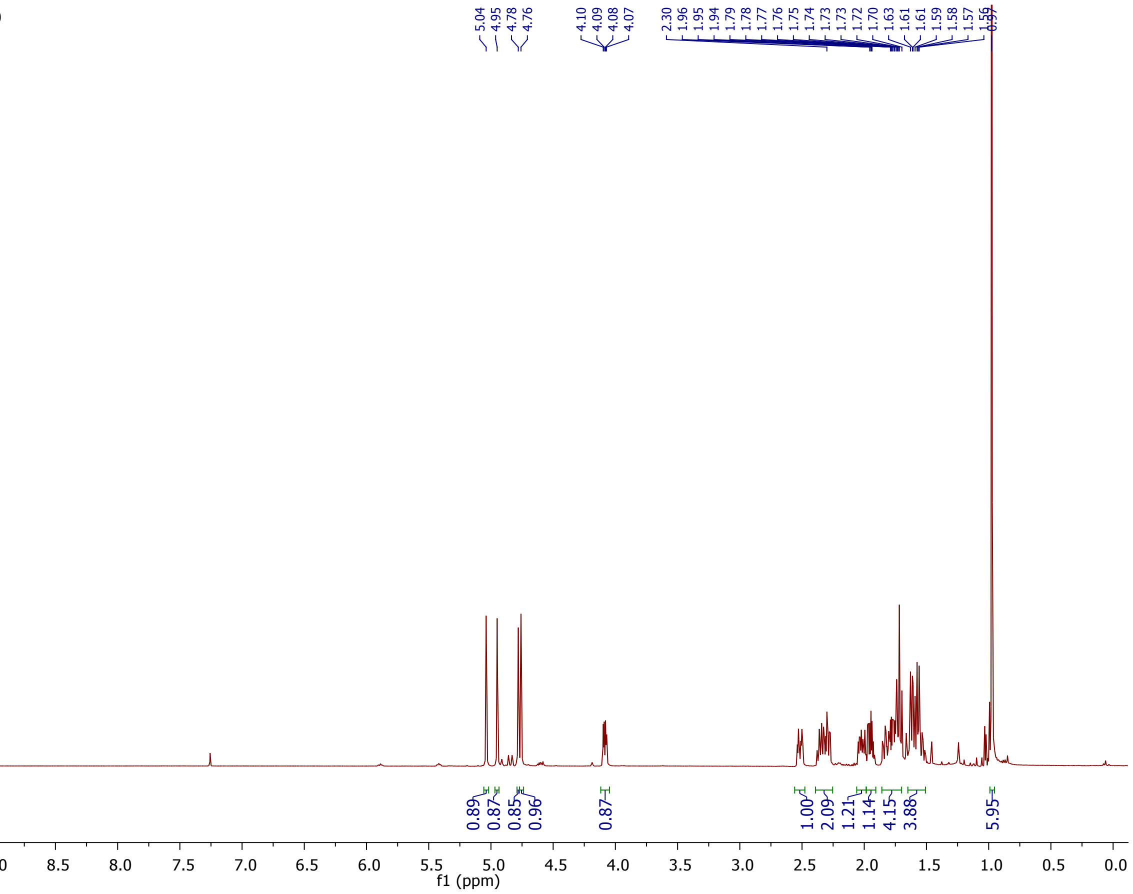

13C NMR (126 MHz, CDCL3)

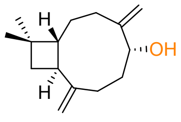

9a

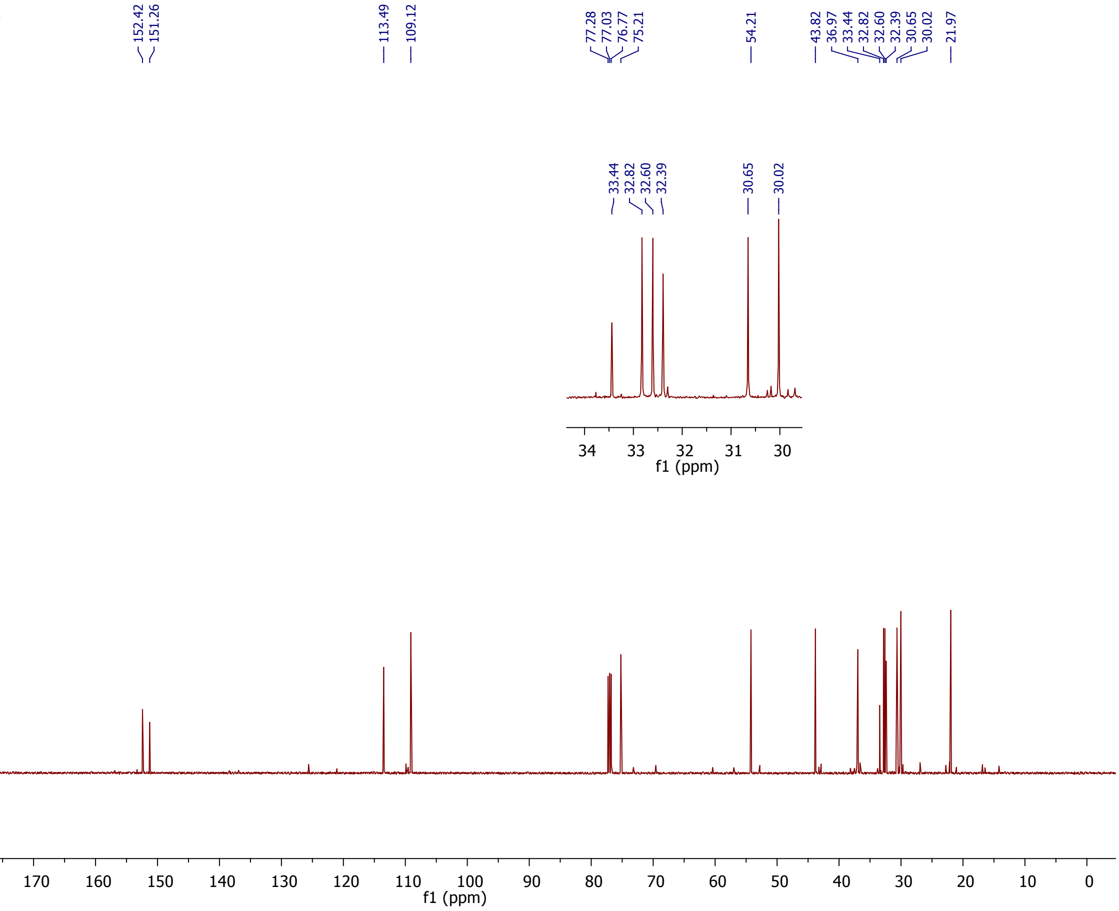

(1*R*,2*S*,5*R*,8*S*,9*R*)-1,4,4-trimethyltricyclo[6.3.1.0^{2,5}]dodecane-8,9-diol (16)

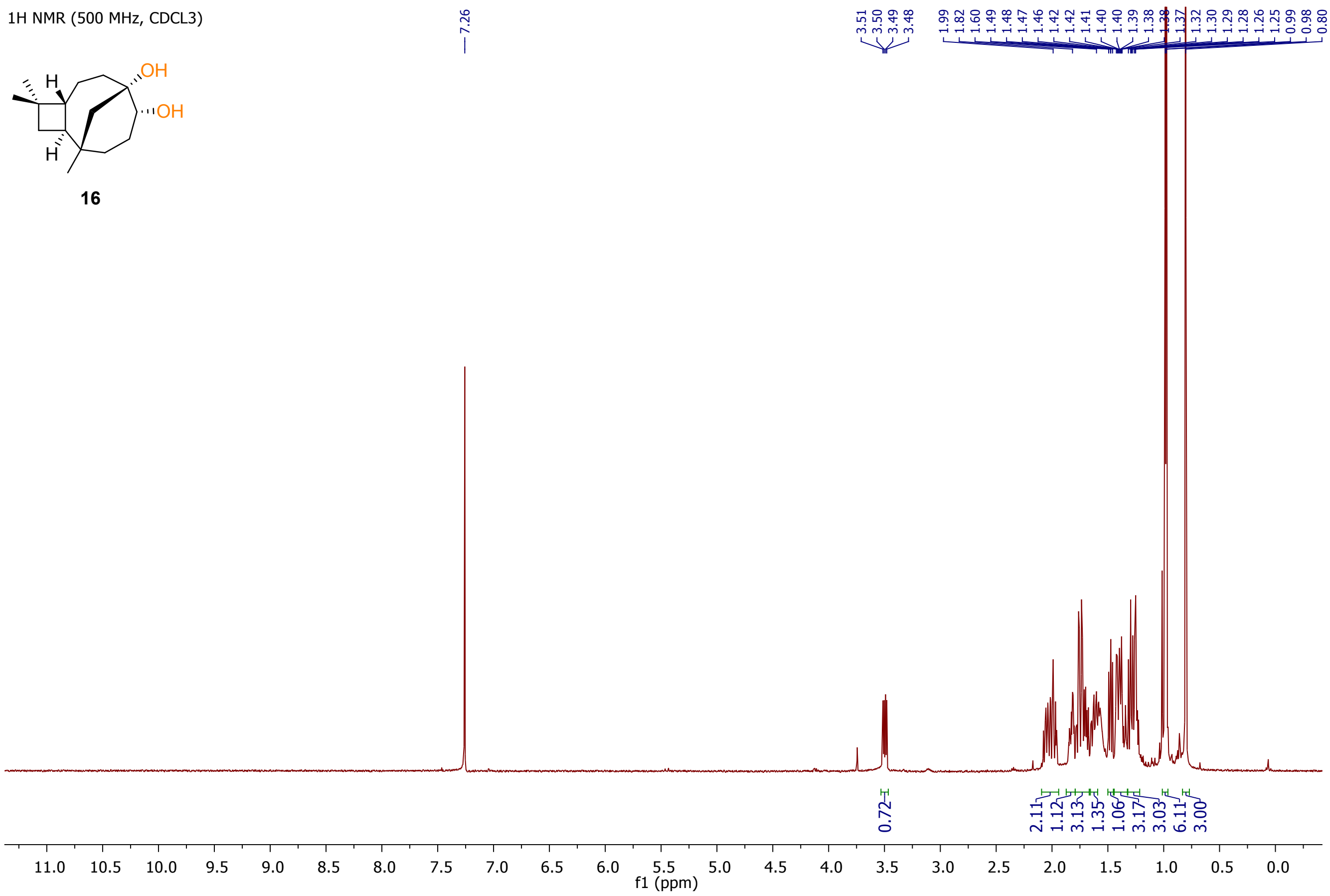

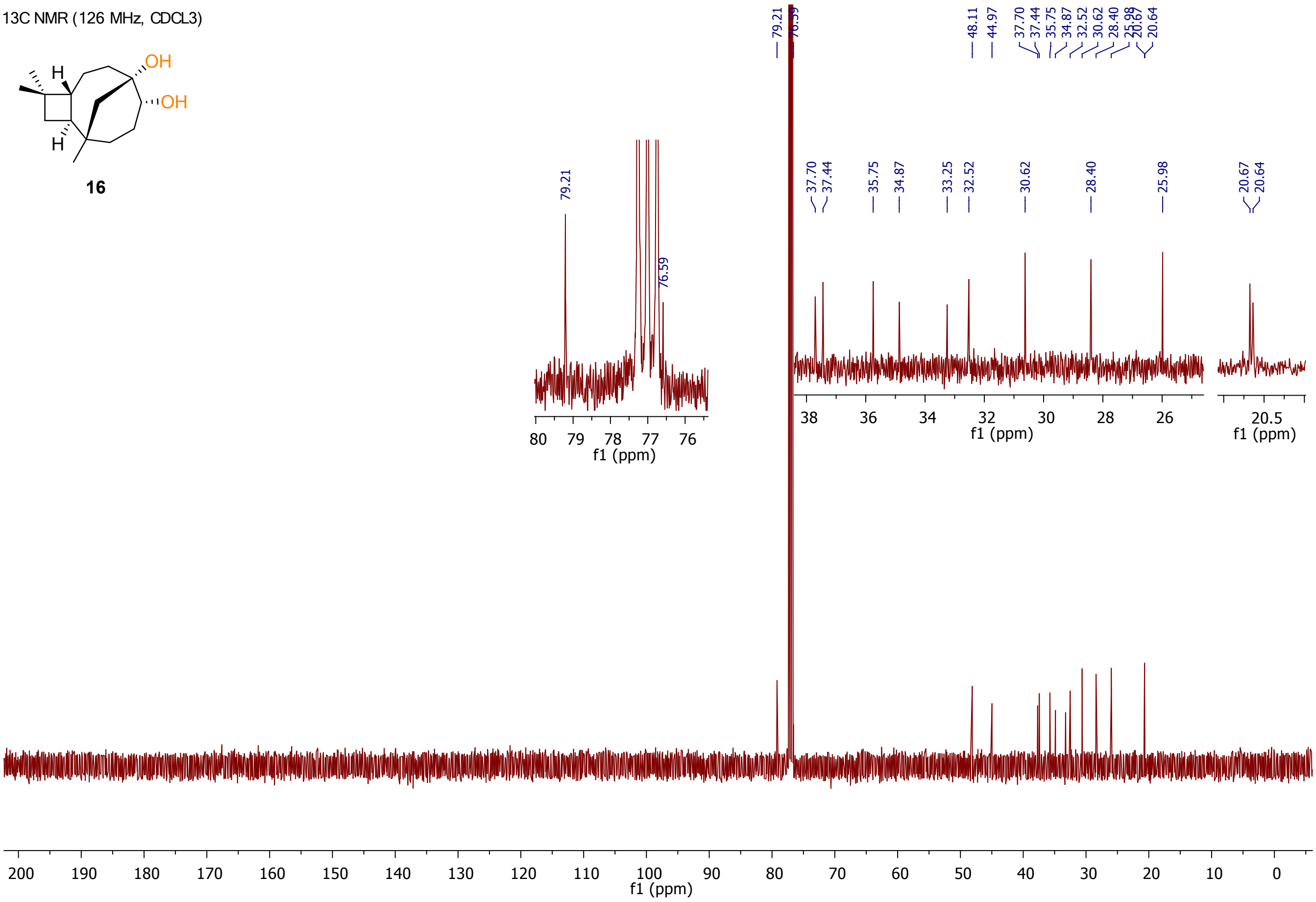

Mixture of isocaryolan-9-one 16a and aldehyde 16b:

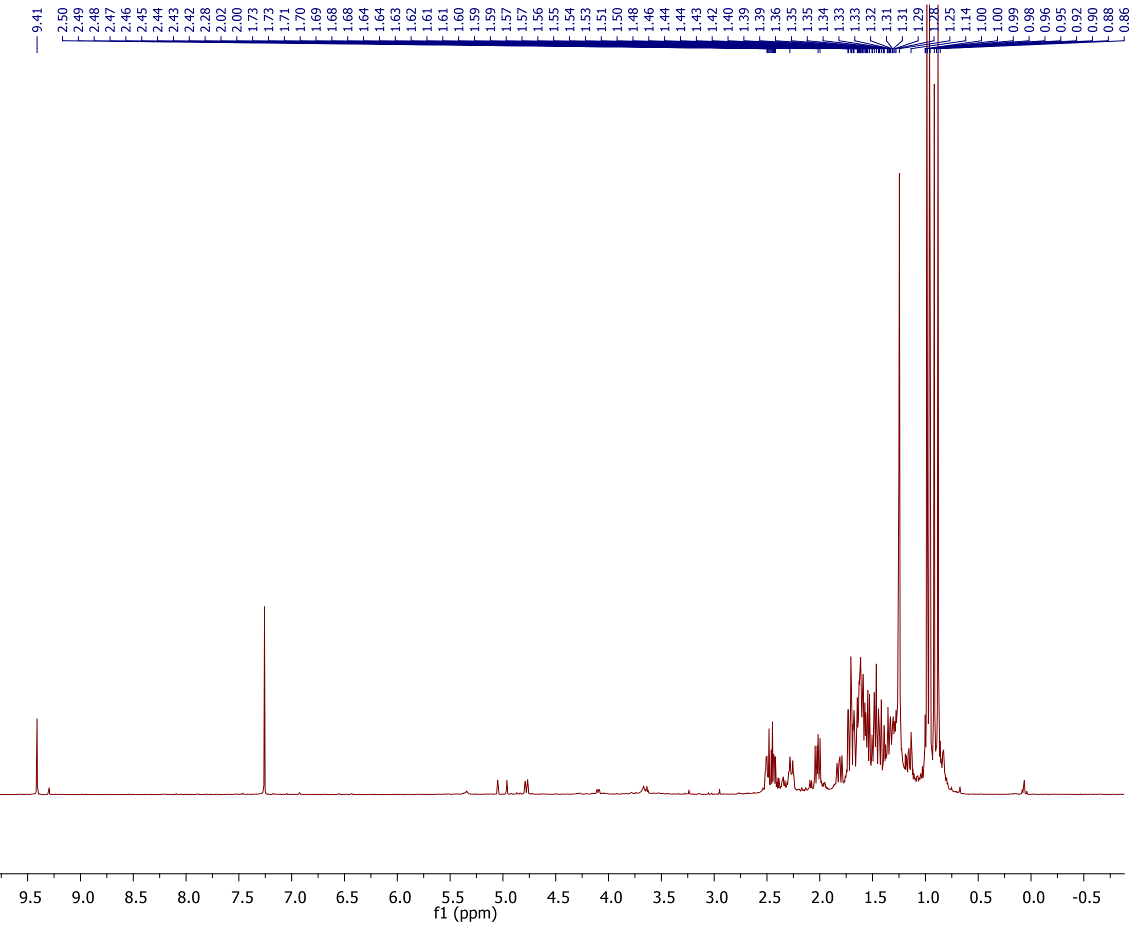

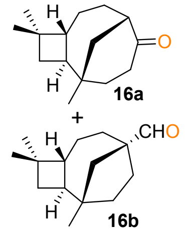

16a + **16b**

3-((1*S*,2*S*,7*R*)-2,8,8-trimethyl-4-oxobicyclo[5.2.0]nonan-2-yl)propanal (17)

1H NMR (500 MHz, CDCL3)

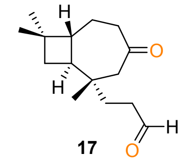

17

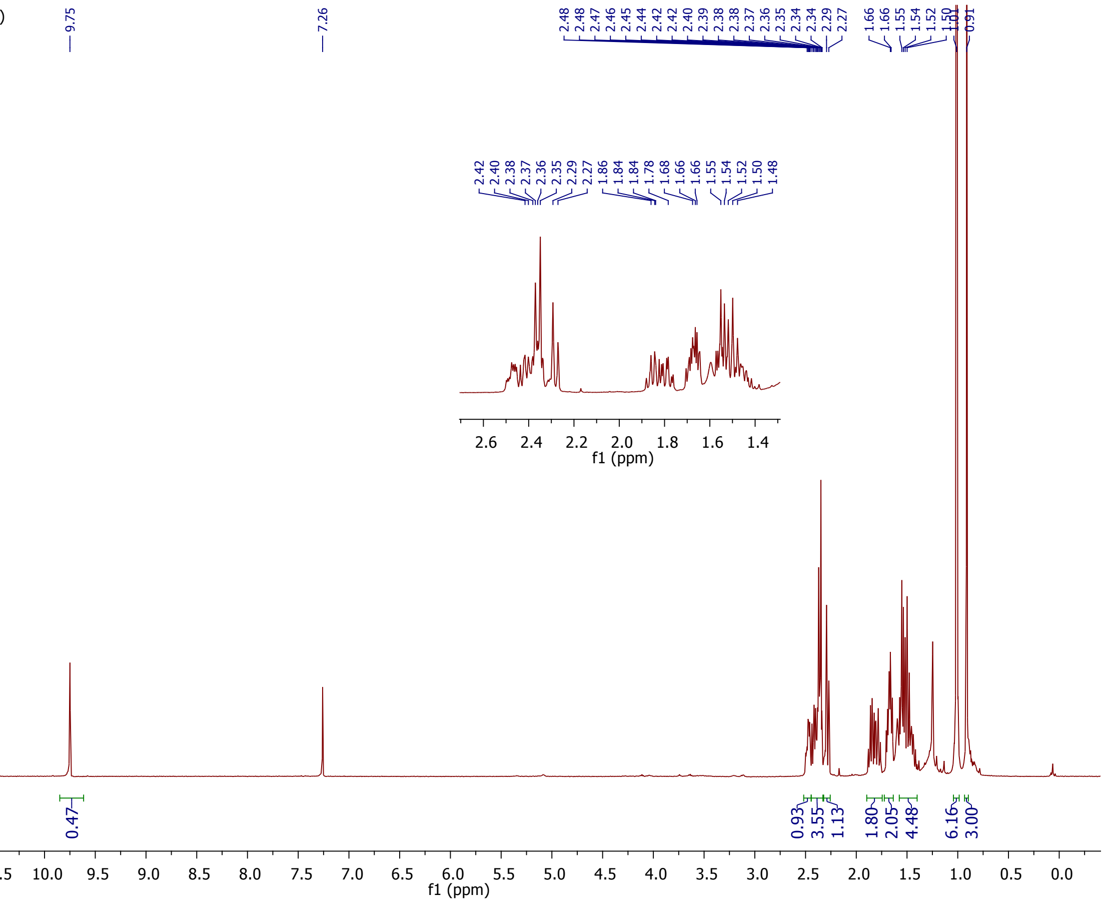

13C NMR (126 MHz, CDCL3)

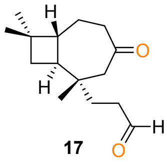

17

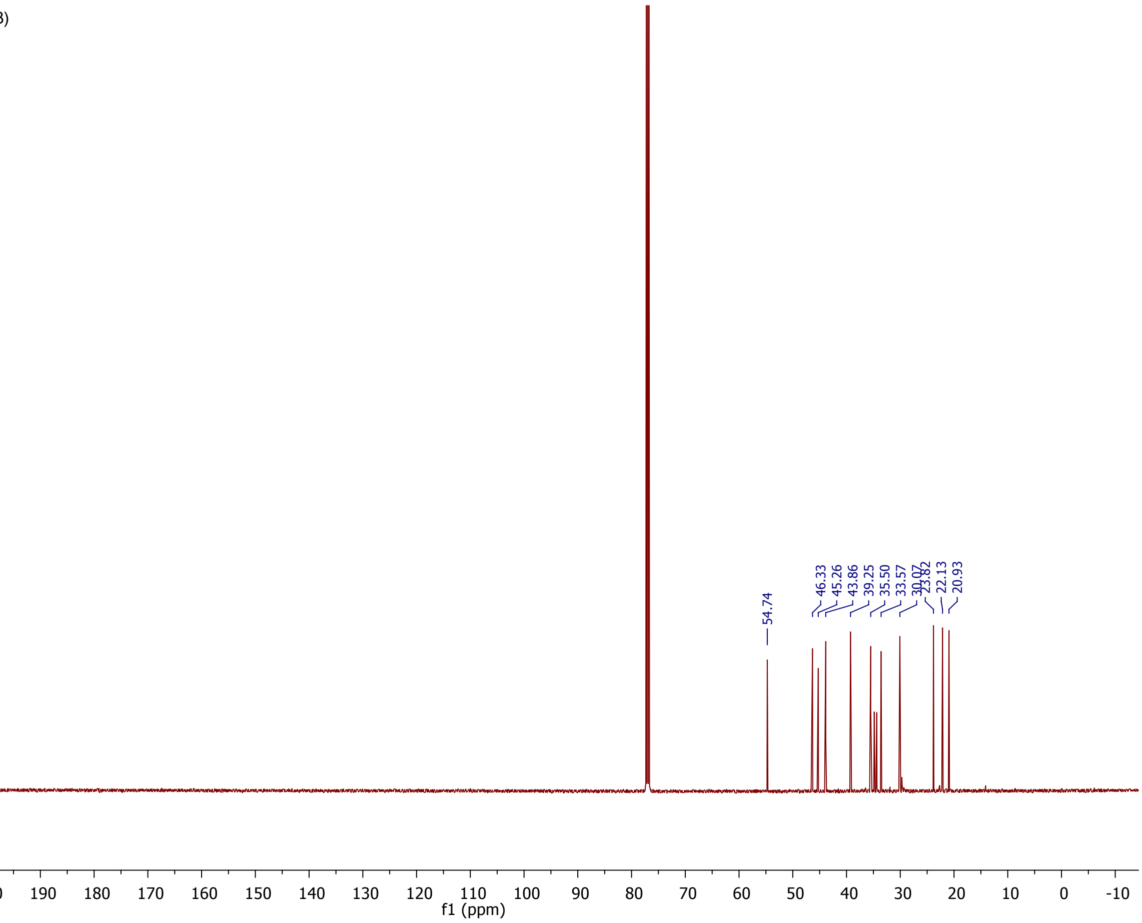

Antipacid A (6)

1H NMR (500 MHz, CDCL3)

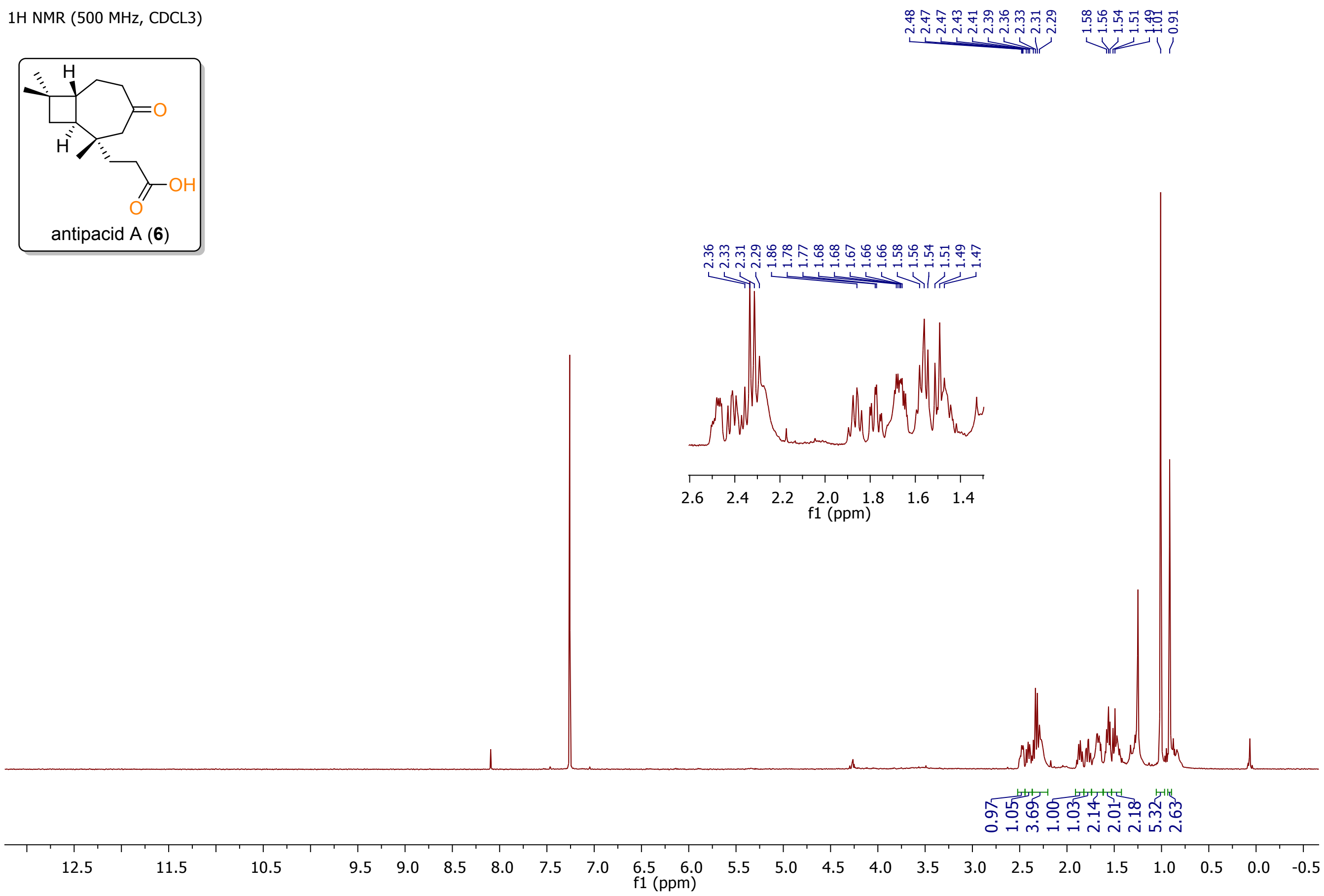

13C NMR (126 MHz, CDCL3)

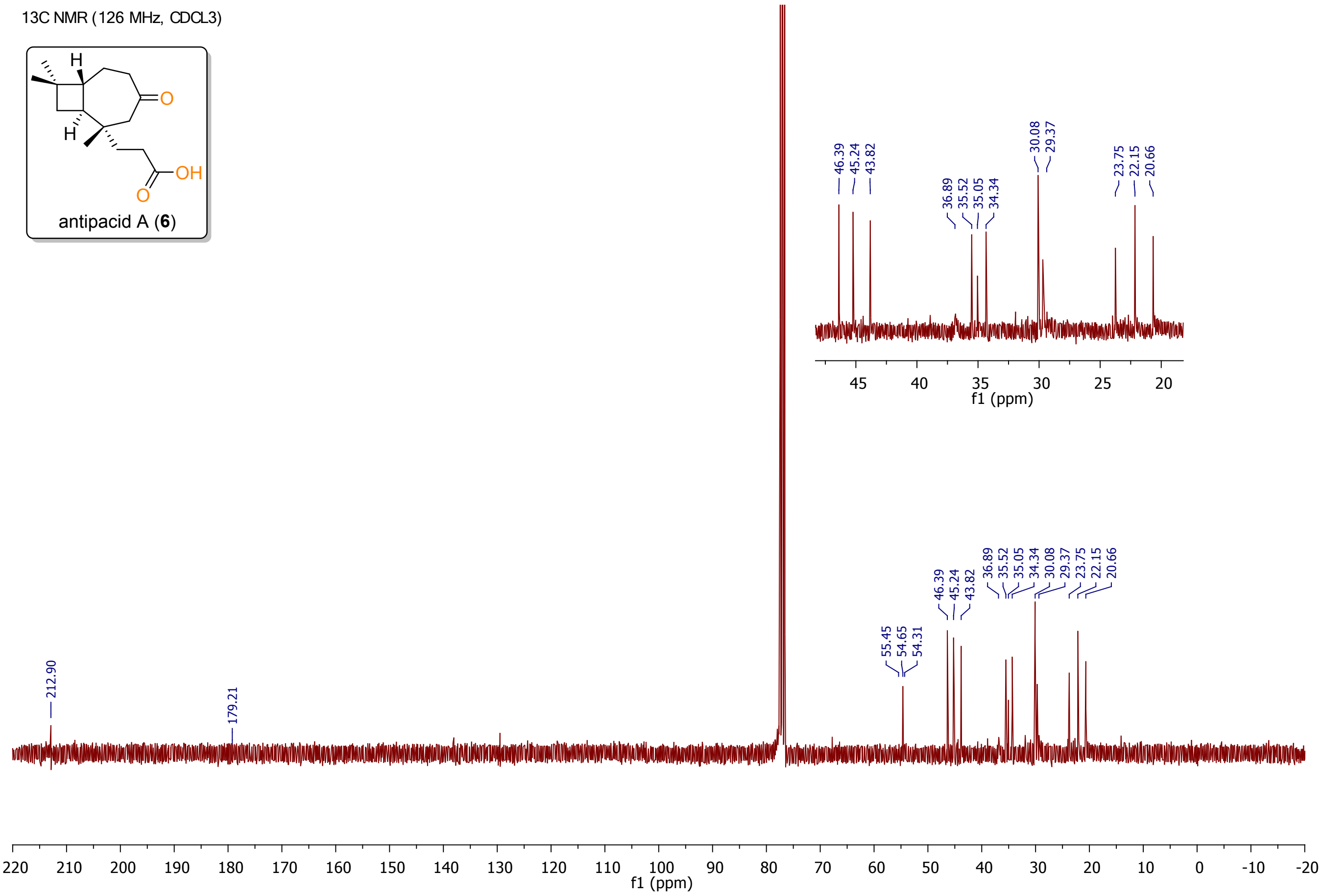

Table S5: Comparison of NMR data of synthetic and isolated antipacid A

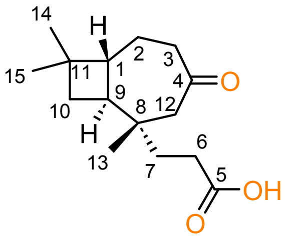

antipacid A (**6**)

**Position**	**This work**	**Isolated***	**This work**	**Isolated***
	**δ_H, type (*J* in Hz)**	**δ_H, type (*J* in Hz)**	**δ_C**	**δ_C, type**
**1**	1.72, ddd (10.4, 10.4, 3.6)	1.77, ddd (10.4, 10.4, 3.6)	45.2	45.3, CH
**2/2'**	1.69, m/ 1.59 -1.60,m	1.70, m/ 1.64, m	23.8	23.7, CH_2
**3/3'**	2.50, m/ 2.43, m	2.48, m/ 2.41, m	43.8	43.8, CH_2
**4**	-	-	212.9	212.8, C
**5**	-	-	179.2	179.1, C
**6**	2.25, m	2.27, m	29.4	29.3, CH_2
**7/7'**	1.69, m/ 1.49-1.52, m	1.72, m/ 1.53, m	36.9	36.5, CH_2
**8**	-	-	35.1	35.0, C
**9**	1.85, ddd (10.4, 10.4, 8.0)	1.87, ddd (10.4, 10.4, 8.0)	46.4	46.3, CH
**10/10'**	1.59-1.60, m/ 1.49- 1.52, m	1.57, dd (10.4, 8.0)/ 1.49, dd (10.4, 10.4)	35.5	35.5, CH_2
**11**	-	-	34.3	34.4, C
**12/12'**	2.32, d/ 2.34**	2.35, d (11.2)/ 2.30, d (11.2)	54.7	54.8, CH_2
**13**	0.91, s	0.92, s	20.7	20.5, CH_3
**14**	1.01, s	1.01, s	30.1	30.1, CH_3
**15**	1.01, s	1.01, s	22.2	22.1, CH_3

*From *Rumphella antipathes* (*Mar. Drugs*, Vol 18, 554, 2020)
** Peak signal is overlapping with peak of protons in position 6
